# A Tiered Multi-Technique Decision-Support Framework for Contaminant Screening and Recycling-Route Assignment of Mixed Plastic Waste

**DOI:** 10.3390/polym18101256

**Published:** 2026-05-21

**Authors:** Aiping Chen, Saumitra Saxena, Vasilios G. Samaras, Bassam Dally

**Affiliations:** 1 Clean Energy Research Platform, Physical Science and Engineering Division (PSE), King Abdullah University of Science and Technology, Thuwal 23955-6900, Saudi Arabia; aiping@mail.ust.edu.cn (A.C.); bassam.dally@kaust.edu.sa (B.D.); 2 Analytical Chemistry Core Lab, King Abdullah University of Science and Technology, Thuwal 23955-6900, Saudi Arabia; vasileios.samaras@kaust.edu.sa

**Keywords:** tiered decision-support framework, mixed plastic waste, contaminant screening, recycling-route assignment, FTIR/DSC/TGA, heavy metals and halogens, non-intentionally added substances (NIAS), feedstock quality control, chemical recycling, UNEP Global Plastics Treaty

## Abstract

Recyclers worldwide face a common bottleneck: incoming mixed plastic bales are chemically opaque, yet the choice between mechanical recycling, chemical recycling, and energy recovery hinges on contaminant levels that cannot be judged by visual inspection alone. This study develops and validates a tiered analytical decision-support framework that translates standard laboratory measurements into explicit, actionable go/no-go routing criteria for any mixed polyolefin waste stream. The framework is organized into three successive analytical tiers of increasing specificity: Tier 1 uses FTIR and DSC for rapid polymer identification and thermal subclass confirmation; Tier 2 applies TGA/DTG for thermal stability assessment and filler quantification; and Tier 3 deploys ICP-OES, WD-XRF, CIC, and TG–MS for targeted heavy metal, halogen, and evolved gas profiling, triggered only when Tier 1/2 flags are raised. This staged logic minimizes unnecessary testing while ensuring that contaminant-relevant information is captured where it matters. The framework is demonstrated on nine blind mixed plastic waste streams (P1–P9) supplied by an industrial recycling facility without prior disclosure of polymer identity, filler content, or additive history—conditions that replicate the uncertainty encountered at any sorting plant globally. Application of the tiered protocol identified dominant polymers (HDPE, LDPE, PP), quantified inorganic fillers (CaCO_3_ up to ~38 wt%), and detected hazardous contaminants, including chlorine (up to ~1900 ppm), lead, chromium, and titanium, enabling each stream to be assigned to a specific recycling route with defined contaminant thresholds. Because the method relies exclusively on commercially available, vendor-independent instrumentation and follows a reproducible, rule-based decision logic, it is directly transferable to recycling facilities in any geographic context without site-specific calibration. The proposed framework thus provides a practical, scalable decision-support tool for feedstock-level quality control under emerging regulations such as the UNEP Global Plastics Treaty.

## 1. Introduction

Plastics have become indispensable in modern life, yet the sheer volume of plastic waste now poses a global crisis. According to the Plastics Europe 2025 industry report “Plastics—the Fast Facts,” global plastics production reached approximately 430.9 Mt in 2024 [1]. A figure that continues to rise year-on-year [2,3], yet global plastic recycling rates remain stagnant at approximately 9% [4] (OECD Global Plastics Outlook, 2022; confirmed by the 2026 update) [4,5], while the vast majority is landfilled, incinerated, or leaks into the environment [3,6]. This imbalance has resulted in widespread pollution—an estimated 70% of used plastics end up as litter or in dumpsites, contaminating oceans, rivers, and soils [6]. Plastics persist for decades, and their fragmentation into microplastics and nanoplastics enables bioaccumulation across trophic levels, including humans. Recent studies have detected microplastic particles in human blood, lungs, and even placental tissue, raising new concerns about chronic exposure and toxicological risks [7,8]. Such mismanaged plastic waste is fueling environmental and health concerns: it accumulates as marine litter and microplastics, threatens wildlife, and even infiltrates the human food chain [9,10,11,12]. Moreover, the plastics life-cycle contributes significantly to climate change, accounting for roughly 3.4% of global greenhouse gas emissions [4]. These multifaceted impacts underscore the urgency of improving global plastic waste management.

Policymakers worldwide are responding to the challenge of plastic pollution with ambitious frameworks and regulations. Plastic waste reduction is now interwoven with international sustainability agendas, including the United Nations Sustainable Development Goals (notably SDG 12 on responsible consumption and SDG 14 on marine conservation). In March 2022, the United Nations adopted a historic resolution to negotiate a legally binding Global Plastics Treaty by the end of 2024 [13,14,15,16]. This treaty, in its current draft, takes a life-cycle approach, aiming to curb new plastic production, phase out the most hazardous plastic products, and address pollution from all plastic sources, including microplastics and toxic additives. Concurrently, regional initiatives have gained momentum. Under the European Green Deal, for example, the EU is strengthening legislation to reduce plastic waste—implementing EU-wide bans on many single-use plastics, imposing new rules to cut packaging waste, and targeting microplastic pollution [17]. Dozens of nations have likewise enacted policies such as plastic bag bans, product take-back mandates, and recycled content standards. As of 2024, over 120 countries have introduced partial or total bans on single-use plastic items (like shopping bags) or related fees to discourage their use [18,19]. These collective efforts reflect a growing global consensus that decisive action is needed to rein in plastic pollution and foster a circular economy for plastics.

Despite these policy advances, formidable scientific and technical challenges remain [13,20]. Plastics represent a very large group of individual polymers with varying chemical and technical characteristics [21], making plastic waste one of the most complex material mixtures from a recycling perspective [22]. The most widely used polymers include polyethylene terephthalate (PET), high-density polyethylene (HDPE), low-density polyethylene (LDPE), polyvinyl chloride (PVC), polypropylene (PP), and polystyrene (PS) [22,23]. Additionally, ‘enhanced’ polymers, known as ‘engineered polymers,’ such as acrylonitrile butadiene styrene (ABS), polycarbonate (PC), and polyurethane (PUR), are also manufactured. ‘Pure’ plastics often exhibit high degradability along with poor thermal, mechanical, and/or esthetic properties, necessitating specific chemical adjustments (e.g., plasticizers, antioxidants, and/or stabilizers) to achieve the desired functionality [24,25].

One critical bottleneck is the heterogeneous and chemically complex nature of post-consumer and post-commercial plastic waste streams, which often include legacy additives [23,24,25], fillers [26], pigments [27], degradation products [28,29,30], and non-intentionally added substances (NIAS) [31,32]. Recent studies have identified over 10,000 chemical substances in plastics [33], many of which are either inadequately regulated or entirely unknown, and several hundred have been found to migrate from recycled plastics into food, water, or the environment [34,35,36,37,38,39,40]. NIAS and additive residues include endocrine-disrupting compounds (e.g., phthalates, bisphenol analogs) [39,41], heavy metals (e.g., lead, cadmium, chromium) [42], and persistent organic pollutants such as chlorinated paraffins [43] and flame retardants [3,25,44], which can leach out or transform under thermal processing, especially during recycling. Alarmingly, recent analyses of recycled plastic pellets from around the world found hundreds of residual toxic chemicals, including pesticides and pharmaceuticals, in recycled material [31,36,45,46,47,48,49]. These findings suggest that recycled plastics can carry a “chemical legacy” of prior use, contamination, or inadequate purification, rendering them unsuitable for sensitive applications such as food packaging or consumer goods. This chemical burden is not merely a quality issue but a public-health and environmental hazard, particularly when recycled plastics are exposed to heat, pressure, or aqueous conditions that may mobilize these substances. Many recent studies note that obtaining accurate, real-time compositional information on mixed plastic waste is essential for proper sorting and recycling [50].

### 1.1. Post-Consumer, Post-Industrial, and Post-Commercial Plastic Waste Types

A further complication is that current research [26,27] has not fully kept pace with the complexity of today’s plastic waste streams. Many studies focus on single-source waste (for instance, analyzing consumer packaging waste or specific industrial scraps in isolation), but relatively few have examined mixed waste streams that integrate post-consumer, post-commercial, and post-industrial plastics together. This represents a significant knowledge gap: without a holistic understanding, we risk optimizing recycling processes for one waste segment at the expense of others. Post-consumer plastic waste (from household use) is often highly diverse in polymer composition and carries a “cocktail” of additives and impurities acquired through product use and disposal. By contrast, post-industrial plastics (industrial scrap or off-cuts) tend to be cleaner and more uniform, making them easier to recycle, while post-commercial waste (from commercial activities) falls somewhere in between. Each waste category differs not only in origin but also in the type and severity of contamination, with significant implications for downstream recycling strategies and contaminant removal processes.

Much of the existing research has focused on clean, single-source waste streams—such as post-consumer packaging, PET bottles, or industrial off-cuts—rather than the real-world mixtures of post-consumer, commercial, and industrial plastic waste typically encountered in material recovery facilities. This segmentation has led to a knowledge gap: without a holistic analysis of integrated streams, we risk designing recycling systems that optimize for one waste class at the expense of others. For instance, post-consumer plastics often carry residues like food waste, inks, or adhesives, requiring more extensive washing and pretreatment, whereas post-industrial plastics may be cleaner but less abundant or homogeneous in polymer type. Post-commercial plastics, such as shrink wrap, retail display materials, or institutional can liners, may share characteristics with both other categories, yet have distinct volumes and collection pathways.

These challenges highlight a critical need for advanced, multi-technique characterization of plastic waste streams to determine their exact polymer composition, contaminant profiles, and degradation signatures. Without such data, accurate sorting, risk assessment, or selection of appropriate recycling routes (e.g., mechanical vs. chemical vs. destruction) is not possible. Many recent studies emphasize the necessity of real-time, high-resolution compositional analysis of mixed waste plastics to support both quality control and regulatory compliance [28,29,30,31,32,33]. To develop effective circular-economy solutions for plastic waste, research must bridge these silos and characterize the full spectrum of plastic waste streams in an integrated manner. To date, such comparative studies remain rare, and a systematic framework for categorizing, analyzing, and valorizing these waste classes is still lacking in the peer-reviewed literature.

### 1.2. Literature Survey

A comprehensive survey of the literature (2015–2026) highlights significant research on characterizing mixed plastic waste streams from post-consumer, post-commercial, and post-industrial sources. The studies summarized in Table S1 (Supplementary Information) demonstrate the extensive application of analytical methods such as FTIR, DSC, TGA, Py-GC/MS, GC×GC-MS, ICP-OES, and XRF to elucidate polymer types, contaminant profiles, and recyclability [25,34,35,36,37,38,39,40,41,42,43,44,45]. These studies span post-consumer municipal waste, specialized streams such as Waste Electrical and Electronic Equipment (WEEE) and Automotive Shredder Residue (ASR), and both mechanical-recycling challenges (e.g., sorting and quality limits) and chemical-recycling or energy-recovery approaches. Each study’s scope (lab vs. pilot vs. model) and focus (polymer identification vs. contaminant characterization vs. thermal behavior) align with different aspects of recycling-system optimization, from plant-level sorting practices to higher-level policy and life-cycle considerations.

Representative studies such as Dahlbo et al. [51], Faraca & Astrup [52], Roosen et al. [53], Beccagutti et al. [54], and Chaine et al. [7] collectively show that contamination (halogens, metals, fillers, and legacy additives) can strongly constrain the mechanical recycling of mixed plastics. Across the literature, recurring gaps include limited integrated multi-technique workflows, insufficient characterization of trace additives/NIAS, and weak linkage between contaminant data and actionable recycling decision thresholds.

### 1.3. Advanced Analytical Approaches for Mixed Plastics

Since 2015, researchers have increasingly employed advanced analytical and multi-technique approaches to better characterize mixed plastic waste streams [55]. Pyrolysis-GC/MS (Py-GC/MS), for instance, has effectively overcome the limitations of traditional IR spectroscopy by accurately identifying and quantifying individual polymers in complex mixtures [56,57,58]. However, translating these laboratory results to real-world waste samples is challenging due to complexities arising from pigments, fillers, contaminants, and varied polymer types, such as PVC and PET. Moreover, Py-GC/MS is a destructive analysis that does not inherently facilitate separation processes, highlighting the current gap in integrating this method into industrial quality control systems for recycled plastics.

The thermogravimetric analysis methods, notably TGA-FTIR and TGA-MS, have become valuable supplementary tools for identifying major polymer types based on decomposition temperatures and characteristic evolved gases. For example, Goedecke et al. [59] demonstrated their utility in distinguishing PVC through characteristic HCl gas emissions or PET via benzoic acid derivatives. Similarly, diffuse reflectance infrared Fourier transform spectroscopy (DRIFTS) has been employed to characterize evolved gases and catalytic degradation products from waste plastics, providing complementary molecular identification of volatiles released during thermal processing [60]. Although beneficial, the full potential of these methods, particularly when coupled with machine learning for automated classification of mixed plastic waste, remains largely untapped [61,62]. Comprehensive two-dimensional gas chromatography coupled with time-of-flight mass spectrometry (GC×GC-TOF-MS) has provided unprecedented resolution and detailed compositional data for complex pyrolysis oils from recycled plastics. Strien et al. [63,64] highlighted its superiority over conventional GC-MS for characterizing highly aliphatic hydrocarbons, which are essential in petrochemical feedstocks. Nevertheless, practical adoption is limited by high costs, complex data interpretation requirements, and the difficulty of translating extensive analytical data into actionable recycling insights.

Emerging directions include machine-learning-assisted spectral classification (e.g., PlasticNet [61]) and rapid elemental screening (e.g., XRF) to support real-time sorting; however, performance on mixed or contaminated streams and scalability to industrial-quality control remain key challenges [61].

### 1.4. Integrating Characterization with Recycling Strategies: Gaps and Outlook

A recurring theme across recent studies is the necessity of comprehensive analytical characterization to enable effective plastic recycling, though several critical gaps remain. One primary gap is the lack of integrated multi-technique analysis: most studies use isolated methods, leading to fragmented data. For example, polymer composition and elemental impurities are often assessed separately. Roosen et al. [53] highlighted that comprehensive studies combining full polymer characterization with quantitative impurity analysis are limited. Future studies must integrate spectroscopic, thermal, and chromatographic techniques to fully characterize waste streams, especially to support emerging recycling technologies such as solvent dissolution or chemical recycling.

Another significant gap is the inadequate characterization of trace additives and contaminants (e.g., plasticizers, UV stabilizers, antioxidants, dyes), which critically affects the quality of recycled plastic. While regulated substances (halogens, heavy metals) are commonly analyzed, many proprietary additives remain overlooked. Dahlbo et al. [51,52] emphasized that detailed chemical composition is essential to ensure recycling quality and limit hazardous substances. Additionally, food residues, oils, and biofilms on plastics—factors that impact recycling quality and necessitate cross-disciplinary analysis involving food chemistry and microbiology—remain understudied. Scaling laboratory findings to industrial recycling operations also poses challenges. Lab-scale analytical results, typically based on small, clean samples, must be translated to large, heterogeneous industrial streams. Although some industrial applications (e.g., XRF scanners at e-waste facilities) successfully sort hazardous fractions, such as brominated plastics, the subsequent recycling or recovery pathways remain unclear or limited, often resorting to incineration rather than material recovery.

A notable disconnect exists between analytical characterization data and practical recycling recommendations. Although many studies broadly suggest improvements in recycling (such as pyrolysis for contaminated plastics or better design for mono-material packaging), actionable details or pilot-scale validations remain rare. Few studies explicitly link analytical data to recycling outcomes, highlighting a need for collaborative interdisciplinary projects where characterization specialists directly inform recycling process trials. Finally, bridging the gap between analytical findings and policy or standardization remains essential. Industry reports (e.g., by BSEF) complement academic findings, highlighting contaminants such as brominated flame retardants in WEEE plastics and informing discussions on regulatory thresholds. However, explicit regulatory standards (e.g., specifying allowable contaminant levels) remain under development. Robust analytical characterization will be crucial to enforce these standards and ensure the safety of recycled plastic.

### 1.5. Integrated Analytical and Recycling Framework for Mixed Plastic Waste

**Figure 1:**
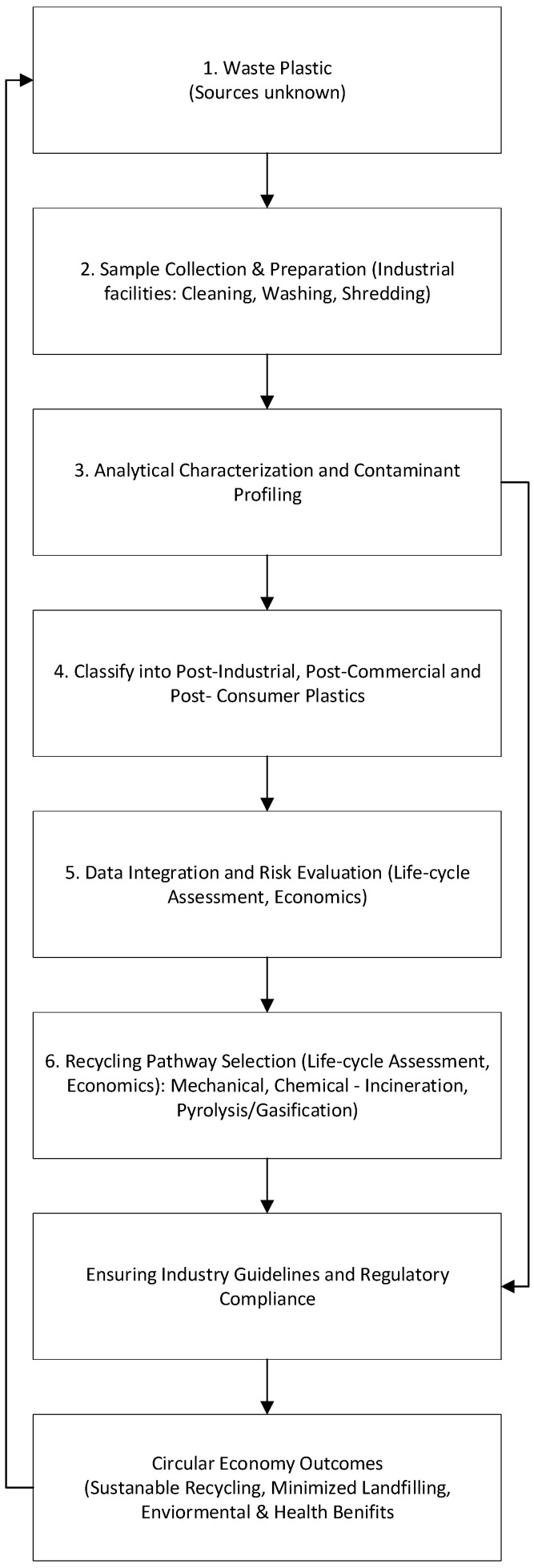
Integrated analytical and recycling framework for mixed plastic waste, showing key stages from sampling to recycling strategy selection. Iterative feedback loops allow refinement based on analytical results, technical feasibility, and regulatory needs, supporting circular economy outcomes.

The framework begins by sourcing plastic wastes from diverse origins—post-consumer, commercial, and industrial streams—collected at industrial recycling facilities (see Figure 1). Unlike many laboratory studies that work with well-defined polymers, the nine samples (P1–P9) were intentionally treated as unknowns upon receipt. This ‘black-box’ approach reproduces the ambiguity recyclers face when bales arrive with incomplete labeling. After initial sample preparation, a comprehensive analytical characterization is conducted using multiple advanced techniques, including spectroscopic (FTIR, Raman), thermal (DSC, TGA), elemental (ICP-OES, XRF), and organic contaminant analysis, as well as migration assessments. The characterization results are integrated into a detailed risk evaluation that identifies polymers, additives, and contaminants and assesses their environmental and health implications. Based on these insights, recycling pathways—mechanical recycling for low-contamination materials, chemical recycling methods such as pyrolysis or gasification for highly contaminated fractions, and necessary pretreatment processes—are selected. Importantly, this stage includes an iterative feedback loop: if new risks or data inconsistencies are identified, further re-analysis or re-evaluation is conducted, refining recycling choices.

Practical recommendations are then formulated for industry stakeholders and regulatory compliance, explicitly aligned with global regulatory frameworks such as the UNEP Plastics Treaty. Another critical source of iterative feedback occurs here: regulatory requirements or industry constraints can prompt additional analytical characterization, adjustments to recycling pathways, or further economic assessments. Ultimately, this iterative and adaptive framework ensures ongoing improvements and alignment with practical and regulatory needs. The holistic integration of advanced characterization, iterative risk evaluation, economic feasibility, and regulatory feedback ensures effective, sustainable recycling practices that meaningfully contribute to circular economy goals and reduce environmental and human health risks associated with plastic waste.

### 1.6. Goals and Objectives of the Present Study

This study aims to bridge critical knowledge gaps by establishing a comprehensive, multi-technique analytical framework for characterizing hazardous contaminants in mixed plastic waste and informing recycling pathway selection. Nine representative plastic waste samples (P1–P9) were analyzed as blind unknowns to mirror real-world feedstock uncertainty, with the primary goal of assessing polymer composition, elemental/halogen contamination, and thermal behavior in order to support practical, source-agnostic recycling decisions.

To achieve this, a suite of analytical techniques—including Fourier Transform Infrared (FTIR) spectroscopy, Raman spectroscopy, Differential Scanning Calorimetry (DSC), Thermogravimetric Analysis (TGA), Thermogravimetry–Mass Spectrometry (TG–MS), Inductively Coupled Plasma Optical Emission Spectroscopy (ICP-OES), Wavelength Dispersive X-ray Fluorescence (WD-XRF), and Combustion Ion Chromatography (CIC)—was applied to identify polymer types, inorganic additives, and halogen contamination across the samples. These methods were further used to assess thermal degradation pathways and material stability, linking decomposition behavior to polymer identity, filler content, and oxidation.

Based on these findings, the study proposes tailored recycling recommendations: mechanical recycling for cleaner, low-additive samples and chemical recycling or energy recovery for heavily contaminated or halogen-rich materials. This feedstock-level assessment also establishes a baseline characterization protocol to support regulatory compliance, including future benchmarks under the UNEP Global Plastics Treaty, and to inform safe, sustainable circular economy practices.

The blind-study results support a screening-based decision tree that plant operators and regulators can apply with a tiered analytical burden when routing mixed plastic waste for recycling or pretreatment.

## 2. Materials and Methods

### 2.1. Plastic Waste Samples (P1–P9)

Plastic waste samples P1–P9 were provided by generic industrial enterprise (Saudi Arabia), which operates advanced recycling facilities in Jeddah and Dammam and is actively advancing circular-economy recycling of plastic scrap. The sample set encompassed a broad spectrum of predominantly post-industrial and post-commercial plastic scraps, including plastic films, clogs, lumps, and non-woven diaper trim. At the recycling facility, incoming materials first undergo manual and/or automated sorting to remove gross non-plastic contaminants (metals, paper, textiles, food residue). The cleaned stream is then sorted and categorized by polymer type (using a combination of near-infrared optical sorting and manual picking) and by color, to preserve resin properties for high-quality recycled applications. Sorted fractions undergo washing to remove surface dirt, labels, and adhesive residues, followed by shredding/granulation to reduce particle size. A density-based separation step (float–sink in water) is used to separate polyolefins from denser polymers (e.g., PET, PVC), and a thickness-based screening step removes oversized or unmolten particles during extrusion. The separated flakes are then dried, compounded in a twin-screw extruder (barrel temperature typically 180–220 °C for polyolefins), and melt-filtered before pelletization. Plastics are operationally managed across seven main groups to support efficient recycling and contamination control. The exact equipment models, screen sizes, and chemical concentrations used at the Napco facility are proprietary; the descriptions above reflect the general process flow as communicated by the facility operator. Importantly, the samples were supplied to the authors as anonymous ‘blind’ materials without sample-specific provenance (post-consumer vs. post-commercial vs. post-industrial), necessitating a reverse-engineering, multi-technique characterization approach to infer polymer identity, degradation state, and contaminant burdens.

### 2.2. Analytical Methodology for Plastic Waste Characterization: Instrumentation and Experimental Procedures

**Figure 2:**
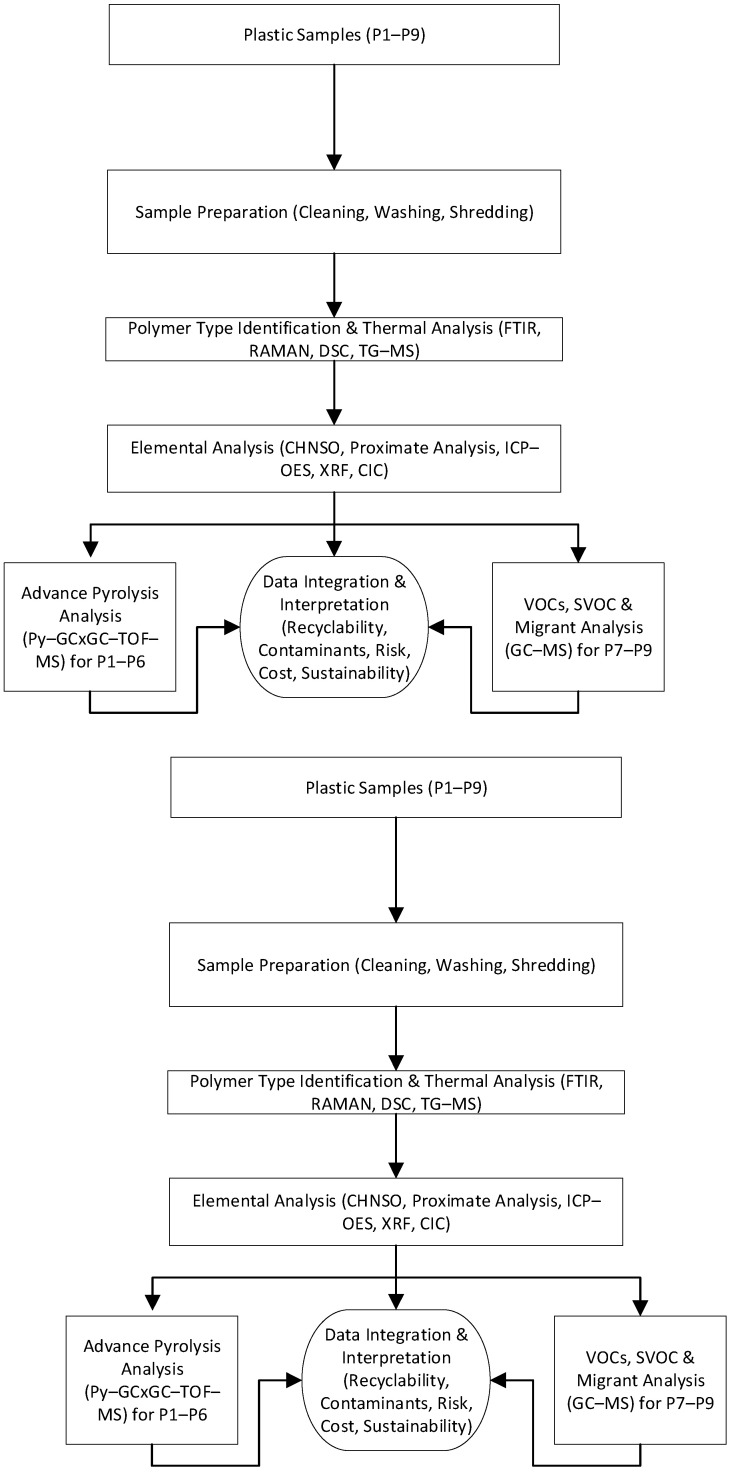
Analytical characterization workflow for mixed plastic waste samples (P1–P9) in this study. Core analyses (FTIR, Raman, DSC, TGA/TG–MS, CHNSO, ICP-OES, WD-XRF, and CIC) were applied to each homogenized sample stream to determine polymer identity, degradation indicators, and contaminant burdens relevant to recycling pathway selection.

An integrated, multi-technique analytical framework was employed to comprehensively characterize nine plastic waste samples (P1–P9) representing post-consumer, post-commercial, and post-industrial origins. The analytical workflow was organized into three core stages: polymer identification and thermal behavior analysis; thermal stability and decomposition profiling; and elemental and halogen quantification (see Figure 2).

To identify polymer types and evaluate thermal transitions, Raman spectroscopy, Fourier Transform Infrared (FTIR) spectroscopy, and Differential Scanning Calorimetry (DSC) were applied. Raman imaging was performed using a WITEC Apyron Raman microscope (Ulm, Germany) equipped with a 473 nm excitation laser and a charge-coupled device (CCD) detector. FTIR spectra were collected using a Thermo Fisher Nicolet iS10 instrument (Waltham, MA, USA) in ATR mode over 500–4000 cm^−1^ at 4 cm^−1^ resolution. Reference spectra for common polymers were acquired and used for spectral comparison (FTIR: polyethylene (PE), PP, PET, poly(methyl methacrylate) (PMMA), PVC, PC, ABS; Raman: PE, PP, PET, PMMA, PVC). Raman identification can be limited by weak scattering and fluorescence in aged/contaminated materials; when present, fluorescence was mitigated by reduced laser power, baseline correction, and cross-validation against FTIR/DSC assignments. DSC analyses were performed on a TA Instrument DSC 250 (New Castle, DE, USA) by heating samples from 30 °C to 300 °C at 10 °C min^−1^ under N_2_ (50 mL min^−1^) to determine melting behavior and crystallinity, supporting LDPE/HDPE/PP discrimination and assessment of thermal history.

Thermal stability and decomposition behavior were investigated using Thermogravimetric Analysis (TGA) and TGA coupled with mass spectrometry (TG–MS). TGA was conducted using a NETZSCH STA-449-F1 instrument (Selb, Germany) under an argon atmosphere from 25 °C to 750 °C at 10 °C min^−1^ to obtain mass-loss profiles and quantify residual ash (TGA residue). For evolved gas analysis, the TGA was interfaced with a quadrupole mass spectrometer (NETZSCH-Gerätebau GmbH, Selb, Germany) (*m*/*z* 2–128) to provide diagnostic identification of key species (e.g., hydrocarbons, H_2_O, CO_2_) associated with individual mass-loss steps. A short time lag between the TGA signal and the MS ion currents arises from the transfer line and was accounted for during interpretation. In practice, a time-lag correction was established using CaCO_3_ as a standard reference by aligning the TGA mass-loss event with the corresponding CO_2_ (*m*/*z* 44) signal. Empty crucible blank runs were measured under the same temperature program to assess background and carryover. TG–MS was used qualitatively for evolved gas identification only; no external quantitative calibration was applied.

Elemental and halogen content was assessed through ultimate/proximate analysis, spectrometric screening, and chromatographic quantification. Ultimate CHNSO analysis was performed on a PerkinElmer 2000 Series II CHNSO Elemental Analyzer (Waltham, MA, USA) (combustion at 975 °C in O_2_; He carrier gas), and oxygen was determined by pyrolysis in He at 1043 °C. Proximate analysis (moisture, volatile matter, ash, fixed carbon) followed ASTM D3172-13(2021)e1 using the STA-449-F1 platform. Elemental screening was performed using wavelength-dispersive X-ray fluorescence (WD-XRF; Bruker S8 TIGER, Karlsruhe, Germany) on granular subsamples as a rapid, non-destructive qualitative screen. WD-XRF calibration and performance verification were carried out periodically by trained laboratory staff following the manufacturer’s standard operating procedures, using certified reference materials to verify elemental response and energy calibration. Given the heterogeneous surfaces and low-level targets in these materials, WD-XRF was used here as a semi-qualitative screening tool rather than the primary quantitative method. For quantitative metals, samples were microwave-digested (Milestone UltraWAVE, Sorisole, Italy) using 5 mL HNO_3_ (69%) and 1 mL H_2_O_2_ (35%) (Sigma-Aldrich), filtered and diluted to 25 mL (Milli-Q), and analyzed by ICP-OES (Agilent 5110, Santa Clara, CA, USA) for Al, As, Ba, Ca, Cd, Fe, K, Mg, Na, P, Pb, S, Sb, Si, Sn, Sr, Te, Ti, Tl, and Zn [65]. ICP-OES measurements were performed in duplicate (*n* = 2) in most cases and are reported as the mean.

Halogen content (Cl, Br, F, I) was determined using Combustion Ion Chromatography (CIC), following standard oxidative combustion procedures. Each solid sample was subjected to high-temperature combustion in an oxygen-rich environment to convert halogens into their respective hydrogen halides (e.g., HCl, HBr). The resulting combustion gases were absorbed in an aqueous solution and analyzed by ion chromatography using a calibrated anion-exchange column.

Quantification was based on external calibration with halide standards. In the KAUST CIC workflow used here, the practical reporting limit was 1 mg/kg for the reported halogens, and non-detects are reported as <LOD. CIC measurements were used as the primary quantitative dataset in this manuscript; selected materials were additionally analyzed by independent third-party combustion-IC laboratories to document detection thresholds and provide inter-laboratory validation (Supplementary Section S1).

Lastly, Gel Permeation Chromatography (GPC) was performed on samples P1–P6 to assess their molecular weight distribution and degradation history.

All plastic waste samples (P1–P9) underwent standardized preparatory procedures, including cleaning (as received), cutting, and shredding. These steps are standard in feedstock characterization because real recycling streams arrive as heterogeneous mixtures of varying shape, size, and thickness; reducing each stream to homogenized fragments ensures that analytical subsamples are representative of the bulk composition rather than biased by surface contamination or a single piece. Such comminution and homogenization are routinely applied in industrial recycling quality control (e.g., ISO 15270:2008 [66] and in published waste characterization studies. It should be noted that some rigid or elastomeric plastics (e.g., thick-walled PVC pipes, cross-linked PUR foams) may resist standard cutting/shredding and could require cryogenic milling; however, the present sample set, consisting predominantly of polyolefin films, flakes, and pellets, was amenable to ambient-temperature comminution. Pelletized or granular materials were cut into small pieces (<1 mg each), mixed thoroughly, and then subsampled for the individual techniques. Due to limited material and the exploratory ‘blind’ nature of the study, most techniques were performed as single determinations on representative subsamples, while ICP-OES measurements were performed in duplicate (*n* = 2) in most cases and reported as the mean. This manuscript presents the results of the core feedstock characterization, polymer identification, and contaminant profiling used to inform recycling pathway selection.

## 3. Results and Discussion

### 3.1. Identification of Waste Plastic Samples (FTIR, Raman, Thermal Analysis)

#### 3.1.1. FTIR Spectroscopy

FTIR is a widely used tool for rapid identification of polymer families and detection of common additives/degradation features through characteristic mid-IR functional-group bands. In this study, sample spectra were compared against reference spectra of common polymers (PE, PP, PET, PMMA, PVC, PC, ABS). Polypropylene (PP) is characterized by CH_3_ bending near 1375 cm^−1^ and CH_2_ bending around 1456 cm^−1^, with C–H stretching near 2841 and 2917 cm^−1^; a peak near 841 cm^−1^ can indicate tertiary carbons in the backbone [67]. Polyethylene (PE) typically exhibits strong absorption around 1464 cm^−1^ (CH_2_ bending), a band near 720 cm^−1^ (CH_2_ rocking), and strong C–H stretching near 2847 and 2914 cm^−1^ [68]. While FTIR can confirm PE, it cannot reliably distinguish LDPE vs. HDPE vs. VLDPE because their spectra are dominated by similar C–H vibrations; therefore, DSC was used to differentiate PE subclasses via melting transitions and crystallinity.

**Figure 3:**
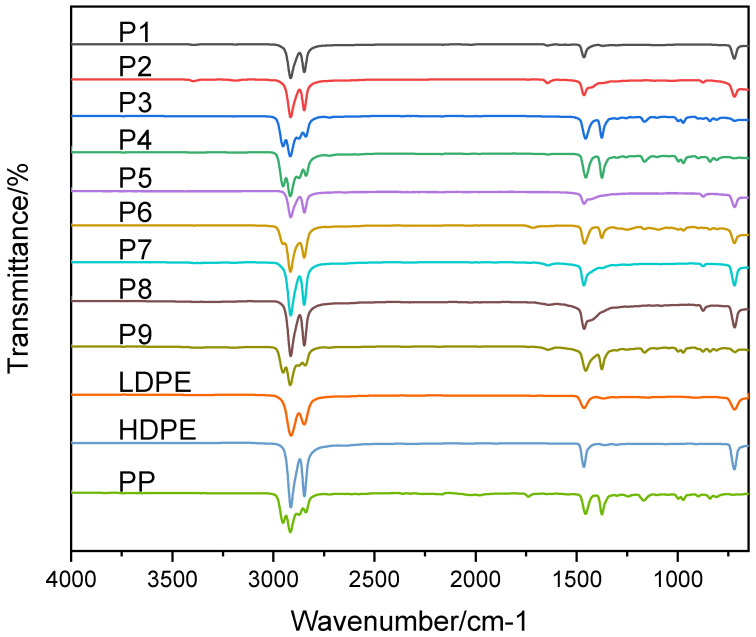
FTIR spectra of plastic waste samples P1–P9, showing characteristic absorption bands for polyolefins. Strong CH_2_ bending (~1460 cm^−1^) and CH_2_ rocking (~720 cm^−1^) indicate polyethylene in several samples (e.g., P1, P5, P7), while CH_3_ bending (~1375 cm^−1^) and broader C–H stretches (~2840–2950 cm^−1^) suggest polypropylene in others (e.g., P3, P4, P9). Peaks near 1715 cm^−1^ and 874 cm^−1^ in select samples indicate oxidation and branching or filler-related features.

Figure 3 presents the FTIR spectra of samples P1–P9 overlaid with standard polymer references. P1, P5, P7, and P8 exhibit bands consistent with polyethylene, including CH_2_ rocking (~720 cm^−1^) and CH_2_ bending (~1460 cm^−1^). P3, P4, and P9 show polypropylene signatures (CH_3_ bending ~1375 cm^−1^; C–H stretches ~2840–2950 cm^−1^). P6 shows overlapping CH_2_/CH_3_ bands, consistent with a PE–PP blend. Several samples show weak features in the carbonyl/unsaturation region (~1715 cm^−1^ and/or ~1645 cm^−1^) that may reflect minor oxidation/weathering, unsaturation, or additive-related functionality; to support comparability, an apparent carbonyl index was calculated from the ATR-FTIR spectra as A1714/A1466 [69] and is reported in Supplementary Table S2.

In summary, the FTIR analysis confirms that the polymers in the nine samples are predominantly polyethylene (HDPE/LDPE) and polypropylene, with variations arising from additives, oxidation, or blending. P9’s more complex spectrum, relative to the clearer PP signatures of P3 and P4, likely results from co-monomer incorporation, additive residues, or greater environmental aging.

#### 3.1.2. Raman Spectroscopy

Raman spectroscopy is a complementary technique to FTIR for identifying polymers, providing vibrational fingerprints based on inelastic light scattering. In this study, reference spectra of standard polymers (PE, PP, PMMA, PVC, and PET) were used for comparison, and the Raman spectra of waste samples P1–P6 were compared to these standards to support polymer identification established via FTIR/DSC (see Figures S2 and S3, Supplementary Information).

The reference PE spectrum matched well with samples P1, P2, and P5, confirming their classification as polyethylene. Similarly, the characteristic peaks of PP were observed in sample P3, consistent with its identification as polypropylene. The PMMA and PVC standards showed distinct spectral features absent in any of the samples, indicating that none of the waste materials matched these polymers. Sample P4 could not be conclusively identified due to a weak Raman signal, likely caused by sample opacity or fluorescence. Sample P6 also exhibited strong fluorescence interference, a phenomenon often associated with the presence of additives, degradation products, or pigments, which absorb and re-emit light and obscure the Raman signal. As a result, P6 could not be conclusively identified by Raman either.

Compared with FTIR, Raman spectroscopy provided consistent polymer assignments when adequate Raman scattering was observed. However, Raman and FTIR have limited ability to distinguish polyethylene subtypes (HDPE vs. LDPE) because these variants share highly similar CH_2_ vibrational bands; therefore, PE subtype differentiation was based primarily on DSC melting transitions rather than vibrational spectra alone [70]. Under 473 nm excitation, selected materials can exhibit fluorescence or weak scattering that may obscure diagnostic bands; common mitigation strategies include longer-wavelength excitation (e.g., 785/1064 nm), reducing laser power, photobleaching, and baseline correction [71]. In this work, Raman was used as a complementary technique alongside FTIR/DSC, and polymer assignment relied on multi-technique consistency, with Raman spectra affected by fluorescence or low signal intensity.

### 3.2. Thermal Analysis

Thermal characterization of the plastic samples was carried out using DSC, TGA/derivative thermogravimetry (DTG), and TG–MS. These methods provided complementary insights into polymer identity, filler content, degradation pathways, and thermal stability. DSC was used to determine melting behavior and crystallinity. Samples showed characteristic melt transitions for polyethylene (LDPE/HDPE) and PP, which supported FTIR- and Raman-based polymer identification. In particular, DSC helped differentiate between LDPE and HDPE—polymers that have nearly indistinguishable FTIR/Raman spectra—by detecting differences in crystallinity and melting temperatures. TGA measured mass loss as a function of temperature, offering information on polymer decomposition and residue content. Combined with DTG, which tracks the rate of weight change, this enabled resolution of overlapping degradation events and identification of multi-step decomposition patterns, often associated with fillers or additives. TG–MS provided real-time detection of evolved gases during decomposition, linking specific weight-loss stages to volatile species. This was especially valuable for identifying CO_2_ release from CaCO_3_ fillers (in samples like P2 and P5), water from oxidized polymers, and hydrocarbon fragments (C_2_/C_3_) indicative of PE or PP backbones. These results not only reinforced the polymer assignments made via FTIR/Raman but also revealed signs of environmental degradation or additive presence that were not evident in the spectra. This integrated approach was critical for assessing the recyclability of each sample and determining whether mechanical or chemical recycling routes would be more appropriate.

**Figure 4:**
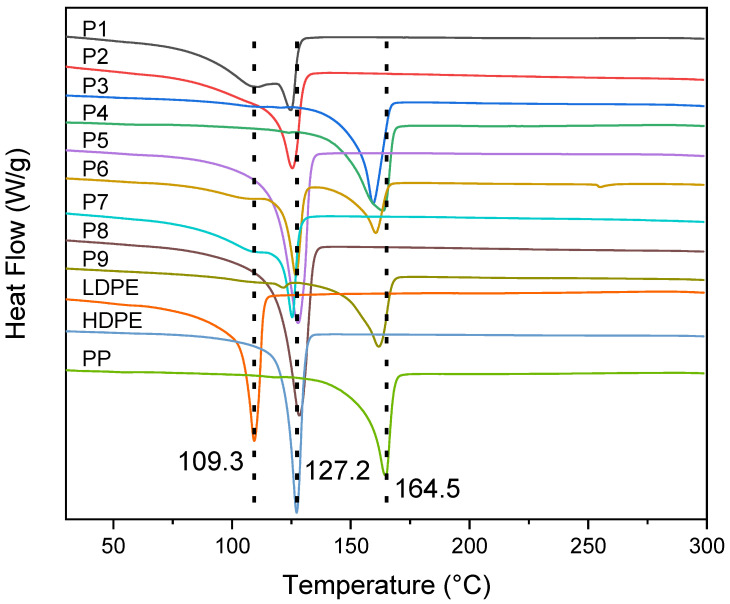
Differential Scanning Calorimetry (DSC) thermograms of plastic waste samples P1–P9. Endothermic peaks near 125–135 °C indicate HDPE-rich compositions, while melting transitions around 160–165 °C correspond to polypropylene (PP). Broader or dual melting peaks in samples such as P1, P6, and P9 suggest blends of PE and PP or the presence of additives and fillers. Peaks between 109 and 115 °C in P5 and P7 are characteristic of LDPE. Thermal transitions support polymer classification and complement FTIR and TGA results.

DSC: Most samples exhibit clear melting points corresponding to their primary polymer compositions, as revealed by the DSC analysis (see Figure 4). Samples P1, P2, P4, and P8 display melting points around 125 °C to 130 °C, indicating they primarily comprise HDPE. However, the DSC profiles of P1 and P4 show slight broadening in the 105 °C to 115 °C range, suggesting the presence of minor amounts of LDPE. Similarly, P8 shows thermal anomalies at higher temperatures, suggesting the presence of inorganic fillers. In contrast, P3, P6, and P9 exhibit melting points of 160–165 °C, characteristic of PP. While these samples are predominantly PP, minor LDPE contamination is observed in P6 and P9 as small thermal events around 105 °C to 115 °C. Samples P5 and P7, with melting points in the 109 °C to 115 °C range, primarily comprise LDPE. However, DSC broadening at higher temperatures in P5 suggests the presence of fillers or additives. Overall, the main melting peaks correspond to the dominant polyolefin types: samples with melting temperatures around 125–135 °C are rich in HDPE, whereas those melting near 160 °C are characteristic of PP. For instance, P1, P2, P4, and P8 all show a principal melt peak at ~130 °C (indicative of HDPE), often with a minor shoulder around 110 °C from a small LDPE fraction. Conversely, P3, P6, and P9 exhibit melting around 160 °C (typical of PP) with slight endotherms near 110 °C due to residual PE. Samples P5 and P7 display melting in the 109–115 °C range, consistent with LDPE. Notably, some DSC curves are broadened or show additional features (e.g., in P5 and P8), suggesting the presence of fillers or additives that affect the thermal transitions.

TGA and DTG: The TGA further confirms the primary polymer compositions and provides additional information on the presence of fillers or contaminants (see Table 1). P1, P3, P4, and P6 show single-stage degradation with onset temperatures around 350–420 °C and T_max between 450 °C and 500 °C, confirming their compositions as relatively pure HDPE or PP, respectively. The total mass loss for these samples is close to 99%, indicating near-complete degradation of the polymer with no significant residue. For these samples, TG–MS detected only the expected hydrocarbon fragments (C_1_–C_3_ gases) and no notable CO_2_ release, consistent with the absence of inorganic fillers. However, P2, P5, P7, P8, and P9 exhibit two-stage degradation patterns. The primary degradation occurs between 350 °C and 500 °C, consistent with decomposition of the polymer matrix, while a secondary mass-loss event between 600 °C and 700 °C suggests the presence of thermally stable additives or fillers. This second mass loss, though smaller (ranging from ~1% to 4%), indicates inorganic materials such as calcium carbonate (CaCO_3_) or carbon black that decompose or oxidize at higher temperatures. In particular, P5 and P8 display more pronounced secondary weight losses, suggesting a higher filler content in those samples. Indeed, TG–MS later confirmed that these high-temperature weight-loss events are associated with CO_2_ evolution (*m*/*z* 44) from CaCO_3_ fillers in samples like P2 and P5.

**Figure 5:**
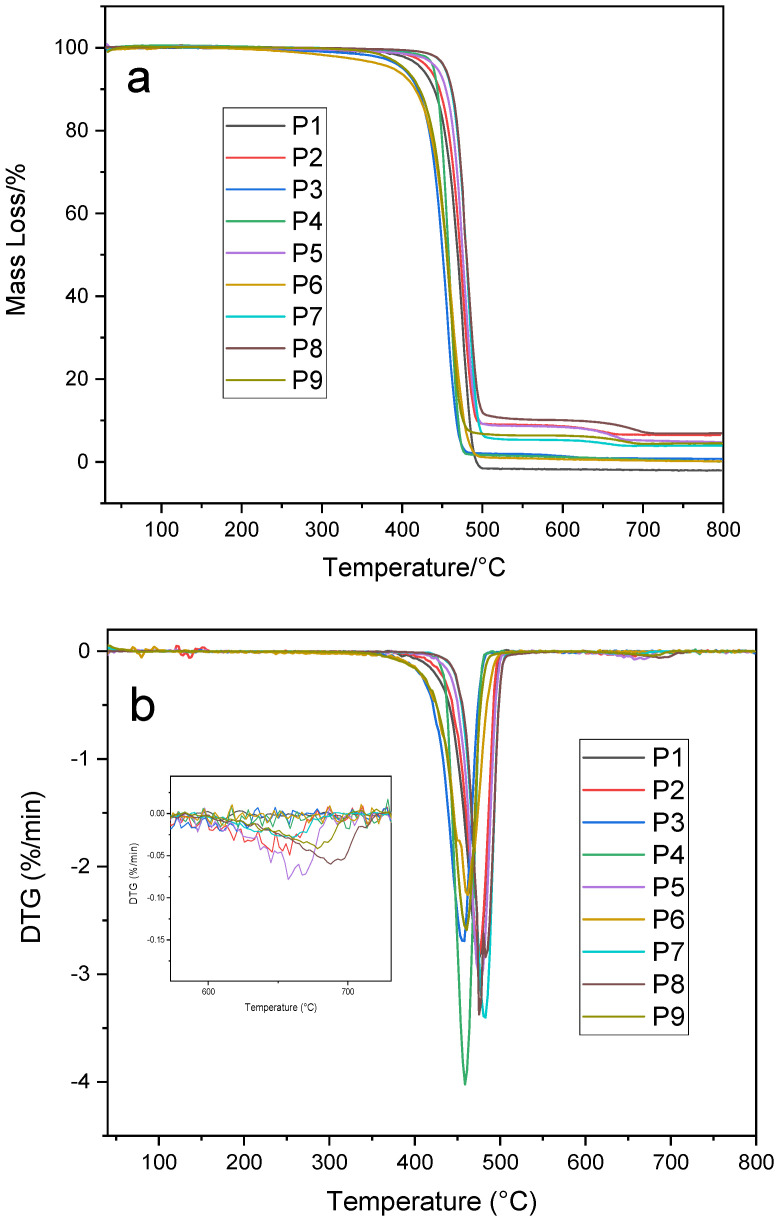
(**a**) Thermogravimetric analysis (TGA) curves of plastic waste samples P1–P9. Most samples exhibit a sharp single-step weight loss around 450–480 °C, typical of polyolefins. Samples P2, P5, and P8 show additional mass loss above 600 °C, indicating the presence of thermally stable fillers such as calcium carbonate. Residual mass varies, reflecting differences in inorganic content across samples. (**b**) DTG curves. Most samples exhibit a primary degradation peak between 450 and 480 °C, characteristic of polyolefins. Secondary mass-loss stages above 600 °C in samples such as P2, P5, and P8 indicate the presence of thermally stable fillers (e.g., CaCO_3_). DTG profiles help resolve overlapping degradation events, confirming polymer purity or the presence of additives.

TGA results revealed two distinct thermal decomposition behaviors among the samples (Figure 5 and Table 1). Most “pure” polymer samples (P1, P3, P4, P6) undergo a single major mass-loss event starting around 350–400 °C and peaking at ≈450–480 °C, with nearly complete weight loss (~99%) and minimal residue. This one-step degradation indicates that those samples are essentially neat polyolefins with little inorganic content. In contrast, samples P2, P5, P7, P8, and P9 exhibit a second, smaller weight-loss stage at higher temperatures (roughly 600–700 °C), accompanied by higher residue, suggesting the presence of thermally stable fillers/additives. In particular, P2 and P5 show pronounced secondary mass losses (~2–4% around 650–700 °C), correlating with significant inorganic filler content. Evolved gas analysis confirms that this high-temperature step is due to CaCO_3_ filler decomposition, which releases CO_2_ [72]. (Calcium carbonate typically calcines in this range, yielding CO_2_ and leaving CaO, consistent with the ≥5% ash residues in these samples). By contrast, the one-step samples show no CO_2_ release and leave <1% ash. The main decomposition temperatures for all samples (~450–480 °C T_max_) are in line with reported thermal stability ranges of polyolefins [65]. Notably, PP-rich samples tend to degrade at slightly lower temperatures (≈440–460 °C) than PE-rich ones (≈460–475 °C) [65], reflecting PP’s lower thermal stability due to its highly branched structure (tertiary C–C bonds cleave more readily) [73]. For example, the PP-based P3 shows a DTG peak at ~458 °C, whereas the HDPE-dominated P1 peaks at ~475 °C; a mixed PP/PE sample like P6 falls in between (~462 °C). XRD of the ash was not performed; carbonate filler presence was inferred by combining elevated TGA residue (ash), Ca enrichment in elemental analysis, and coincident CO_2_ evolution (*m*/*z* 44) in TG–MS.

The DTG profiles precisely identify the degradation rates and stages (see Figure 5). For samples P1, P3, P4, and P6, the DTG curves show a single sharp peak, indicating one-step degradation, confirming that these are relatively pure polyolefins with minimal contamination or filler. On the other hand, P2, P5, P7, P8, and P9 exhibit multiple peaks in their DTG curves. The first DTG peak, occurring around 450 °C to 500 °C, represents the main polymer degradation, while a second, smaller peak around 600 °C to 700 °C aligns with the decomposition of fillers or additive residues. This secondary DTG peak is most pronounced in P5, P7, and P8, supporting the conclusion that these samples contain significant inorganic filler content. In summary, samples P1, P2, P4, and P8 are primarily HDPE (with P2 and P8 containing notable filler content and P1 and P4 having minor LDPE contamination). P3, P6, and P9 are mostly PP (with P9 containing filler and P6 showing a minor LDPE component). P5 and P7 are predominantly LDPE, with both containing fillers; P5 shows the highest filler content.

TG–MS Analysis of Evolved Gases (Figure S3, Supplementary Information): To further investigate the decomposition behavior and evolved volatiles during heating, we performed thermogravimetry–mass spectrometry (TG–MS) on six representative samples (P1–P6) (see Supplementary Data). In these experiments, each sample was heated from 30 °C to 800 °C at 10 °C/min under an argon atmosphere, while the attached mass spectrometer continuously monitored the evolved gases by tracking ion fragments in the *m*/*z* 2–128 range. This hyphenated technique directly links the mass loss events observed in TGA to specific gaseous products, complementing the TGA/DTG curves with molecular identification of the volatiles. Figure S3 (Supplementary Information) presents the evolution profiles of key ion fragments corresponding to major evolved gases. As shown in the TGA results, all six samples undergo their main decomposition between roughly 350 °C and 500 °C, but with slight shifts reflecting polymer type. PE-based samples P1 and P2 exhibit T_max around ~475 °C, whereas the PP-based samples P3 and P4 show a lower T_max around ~458 °C due to PP’s slightly lower thermal stability. Sample P5 (identified as LDPE) has a primary degradation peak at ~477 °C and, notably, a secondary weight-loss event at ~667 °C. This high-temperature event is attributable to filler decomposition, consistent with the CaCO_3_ content indicated by its two-step TGA profile. Sample P6, a PP/PE blend, displays a decomposition peak at ~462 °C, intermediate between that of pure PP and PE, reflecting its mixed polymer composition. These TGA/DTG trends align with known decomposition ranges for PE and PP polymers.

TG–MS of representative samples (P1–P6) further elucidated the decomposition pathways by identifying key evolved gases. All samples released a suite of C_1_–C_3_ hydrocarbon volatiles upon heating, consistent with the random-chain scission of polyolefins [74,75]. However, the distribution of these fragments varied with polymer composition: PP-derived samples produced relatively more *m*/*z* 41 and 43 (propene and propane), whereas PE-rich samples generated higher *m*/*z* 28 and 29 signals (ethylene and ethane). These trends reflect polypropylene’s tendency to form C_3_ fragments at its tertiary carbons versus polyethylene’s yield of C_2_ fragments from random scission.

Importantly, the TG–MS data corroborate these thermal observations—for instance, the secondary weight-loss in P2 and P5 is accompanied by a surge in *m*/*z* 44 (CO_2_) in the mass spectra, directly confirming the decomposition of CaCO_3_ filler in those samples. Mass spectral analysis of the evolved gases reveals that all samples release a similar suite of low-molecular-weight compounds upon pyrolysis, with dominant ion fragments at *m*/*z* 2, 15, 18, 28, 29, 41, 43, and 44 corresponding to H_2_, CH_4_, H_2_O, C_2_H_4_, C_2_H_6_, C_3_H_6_, C_3_H_8_, and CO_2_, respectively. These are exactly the gases expected from the thermal cracking of polyolefins [75,76]. The relative intensity of these fragments, however, varies with polymer composition. PE-rich samples (P1, P2, P5) produce higher signals for *m*/*z* 28 and 29, indicative of abundant ethylene and ethane evolution, whereas PP-rich samples (P3, P4) generate proportionally more *m*/*z* 41 and 43, attributable to propylene and propane. The mixed-polymer sample P6 emits all of the above fragments, essentially combining the PE and PP gas profiles with an overall fragment distribution centered around ~495 °C. Notably, *m*/*z* 44 (CO_2_) appears as a distinct peak at high temperatures in the spectra of P2 and P5, confirming the thermal decomposition of CaCO_3_ fillers via CO_2_ release around ~690 °C [77]. Likewise, *m*/*z* 18 (H_2_O) is more pronounced in the evolved gas profile of the more oxidized sample P2, reflecting moisture release or the breakdown of oxygenated additives (i.e., NIAS) in that sample. The absence of significant *m*/*z* 64 or 81 signals (which could indicate chlorinated species like HCl or chlorobenzenes) in TG–MS suggests that chlorine-containing gases were either below detection or released at low levels (this is examined more directly via halogen analysis later). Overall, thermal analysis corroborated the FTIR/Raman polymer IDs, quantified filler content (via ash), and did not reveal unexpected materials (e.g., no sign of PET, which would leave a char or distinct decomposition profile).

In sum, the integrated thermal analysis (DSC, TGA, DTG) coupled with evolved gas analysis provides a comprehensive picture of each sample’s composition and thermal stability. These results validate the polymer identifications (PE vs. PP) via their characteristic decomposition products and link secondary weight-loss events to inorganic fillers, information crucial for devising appropriate recycling strategies.

As summarized in Table 2, the major ion signals detected from each sample alongside their chemical assignments and interpretations (also see Figure S3, Tables S5 and S6, Supplementary Information). Across all six samples, the primary pyrolysis products are small hydrocarbons (C_1_–C_3_ alkanes/alkenes) and minor amounts of H_2_O and CO_2_. The PE-derived samples show a clear emphasis on C_2_ fragments, confirming their polyethylene origin, whereas the PP-derived samples exhibit a higher relative yield of C_3_ fragments, confirming their polypropylene origin. The presence or absence of CO_2_ in the TG–MS profile serves as a clear marker for inorganic carbonate fillers in the samples. Furthermore, the TG–MS results help distinguish subtle differences not evident from TGA alone—for instance, the elevated H_2_O signal in P2 indicates absorbed moisture or degradation of additives, which correlates with that sample’s high O content from CHN analysis.

Overall, the integration of TG–MS with conventional DSC/TGA provides a more complete thermal characterization of the plastic waste samples. This combined approach offers molecular-level insight into the thermal decomposition pathways of the samples and supports the development of appropriate recycling and safety strategies.

Molecular Weight Distribution (GPC Analysis).

The results (Table 3) show notable differences in the peak molecular weight (Mp), number-average molecular weight (Mn), and weight-average molecular weight (Mw) across samples. Samples P3 and P4 (PP-rich) exhibited the highest Mn values (76,526 and 56,685 g/mol, respectively), indicating preserved chain integrity and minimal degradation, consistent with their FTIR spectra (absence of oxidation bands) and clean, single-stage TGA profiles. In contrast, P5 showed the lowest Mn (43,309 g/mol) and Mw (235,918 g/mol), aligning with evidence of oxidation (carbonyl peaks in FTIR), CaCO_3_ filler (CO_2_ evolution in TG–MS), and multi-stage thermal degradation. The relatively low Mn values for P1, P2, and P6 also suggest mild oxidative degradation or a mixed chain population. Overall, the GPC data reinforces the thermal and spectroscopic findings, providing molecular-level evidence for processing history and recyclability: samples with higher molecular weights and narrow distributions (e.g., P3, P4) are better suited for mechanical recycling, while degraded or blended samples (e.g., P5, P6) may require pretreatment or chemical recycling routes.

The integrated summary in Table 4 provides a multi-dimensional profile of each plastic waste sample (P1–P9), combining spectroscopic signatures (FTIR, Raman), thermal behavior (DSC, TGA, TG–MS), and compositional indicators (ash content, CO_2_ evolution) to infer material identity and indicators consistent with different waste histories. Because the samples were received as anonymous ‘blind’ materials without a sample-specific chain-of-custody, any discussion of likely origin is necessarily inferential and is used here to indicate consistency with cleaner scrap versus more aged/filled mixed streams. For example, P1/P3/P4 show single-polymer signatures (HDPE or PP), minimal filler/oxidation, and near-complete volatilization during thermal analysis—features consistent with cleaner production scrap. By contrast, P2/P5 show two-stage decomposition with CaCO_3_-related CO_2_ evolution and higher residue, consistent with filled films/packaging fractions, while P7–P9 show moderate inorganic signatures and polymer blends consistent with more heterogeneous commercial streams. This combined analytical approach therefore supports polymer identification and highlights contamination/degradation indicators relevant to recycling-route selection.

### 3.3. Comprehensive Elemental, Trace Metal, and Halogen Profiling

The proximate and ultimate analyses provide a comprehensive picture of plastic composition and thermal behavior. While proximate analysis assesses moisture, volatile matter, fixed carbon, and ash—critical indicators of how materials behave under heat—ultimate analysis focuses on elemental composition (C, H, N, O), which informs energy content and emission potential. Together, these datasets help determine recyclability, environmental impact, and suitability for thermal processing such as pyrolysis or incineration.

#### 3.3.1. Proximate Composition

As shown in Table 5 (and Figure S4, Supplementary Information), all samples exhibit low moisture content, ranging from 0.02% (P8) to 0.20% (P9), indicating minimal water retention, which is ideal for high-temperature processes such as pyrolysis or incineration, as it reduces the energy required for drying. These samples were already mechanically recycled, and drying was part of the process. Most samples display very high volatile matter content, with P1 (99.41%), P4 (98.92%), and P6 (97.00%) being particularly high. This suggests that these plastics decompose primarily into volatile products during thermal processing, leaving little solid residue. Fixed carbon content is generally low, with the highest observed in P8 (2.70%). Fixed carbon represents the solid residue left after volatile matter is driven off, and low values indicate that most carbon will be converted to volatile compounds with minimal char formation. Samples P5 (10.21%), P2 (6.58%), and P8 (4.66%) exhibit relatively higher ash content, indicating the presence of inorganic fillers or contaminants. Higher ash content can pose challenges in recycling and energy recovery, leaving more solid residue after thermal degradation, requiring additional disposal or handling.

#### 3.3.2. Elemental Composition

As shown in Table 6, all samples exhibit high carbon (79.80–86.04 wt%) and hydrogen (12.68–14.03 wt%), consistent with polyolefin-rich plastics and indicative of high energy value. Nitrogen was not detected or present only at trace levels (detection limit 0.1%), suggesting low potential for nitrogenous emissions (e.g., NOx) during thermal processing. Oxygen contents were low (0.61–2.85 wt%), consistent with PE/PP matrices; higher oxygen in selected samples likely reflects partial oxidation and/or oxygen-containing additives or fillers.

Overall, samples P1, P4, and P6—characterized by high volatiles, low ash, and low fixed carbon—are ideal candidates for pyrolysis, producing mainly liquid and gas with minimal residue. In contrast, P2, P5, and P8 contain higher ash and would require additional handling or pretreatment. The low nitrogen and oxygen contents across most samples suggest high energy yield and low emissions risk. Samples P8 and P9, which show slightly higher oxygen and ash levels, may contain fillers or additives that affect recyclability but remain within manageable limits.

#### 3.3.3. ICP-OES

Additives such as stabilizers, pigments, or processing aids may contain metals that can be identified using elemental techniques such as ICP-OES or XRF. Integrating these findings with the polymer assignments established via Raman, FTIR, and DSC helps anticipate how these plastics may behave in recycling streams and whether additional screening or pretreatment is warranted. The ICP-OES results (Table 7) provide a broad view of elemental composition across samples P1–P9, particularly for calcium, iron, sodium, potassium, sulfur, titanium, zinc, silicon, and lead. These elements, present at varying concentrations, can influence suitability for different recycling routes. Halogens such as chlorine were assessed separately by CIC and WD-XRF.

High calcium concentrations in samples P2, P5, P7, P8, and P9 are consistent with the presence of calcium-based fillers, most plausibly CaCO_3_. Detectable iron in multiple samples (notably P2–P5 and P7–P9) points to possible pigment residues, process contamination, or metal-bearing additives. Elevated inorganic loadings can complicate mechanical recycling by affecting product quality and can increase residue generation during thermal processing.

The detection of sodium and potassium in selected samples, particularly the high Na and K levels in P2 and measurable K in P7 and P9, suggests contamination from salts, processing residues, or additive packages. These elements can influence melt behavior in mechanical recycling and, at elevated levels, may also contribute to corrosion concerns in thermal systems. Lead was detected in P7 and P9 at low ppm levels, indicating the need for caution when considering these streams for sensitive recycled-product applications.

Aluminum-based additives are sometimes used in plastics, and while aluminum is generally less problematic for thermal recycling, its presence can interfere with mechanical recycling by reducing product quality. Titanium dioxide (TiO_2_) and silicon-containing fillers, identified in several samples, are commonly used to enhance plastic properties but also increase ash content during thermal processes, complicating residue management.

Arsenic, cadmium, antimony, and tin were not detected in the analyzed samples, while lead was detected only in P7 and P9 at low ppm levels. The non-detection of As, Cd, Sb, and Sn is consistent with the absence—or levels below detection—of several legacy additives such as cadmium pigments, antimony-based flame-retardant synergists, and organotin stabilizers. However, the present dataset should be interpreted as a screening-level analytical assessment rather than a complete regulatory compliance determination.

These screening results are broadly consistent with the reduced use of many restricted additive classes in contemporary materials, but they do not on their own establish full compliance with frameworks such as RoHS or REACH. A formal compliance assessment would require broader targeted testing, traceability, and product-specific regulatory criteria.

#### 3.3.4. Halogen Analysis (CIC)

A dedicated halogen analysis of samples P1–P9 was conducted via combustion ion chromatography to quantify total fluorine (F), chlorine (Cl), bromine (Br), and iodine (I). Table 8 summarizes the halogen concentrations. These elements are crucial to evaluate because halogens, even at low levels, can significantly impact recyclability and environmental safety. WD-XRF served as a rapid screening tool; CIC provides quantitative reporting, and third-party combustion-IC cross-validation is summarized in Supplementary Section S1.

Fluorine (F), bromine (Br), and iodine (I) were below the CIC reporting limit (reported as <LOD) in all analyzed samples summarized in Table 8, except for P2, which was not analyzed because of insufficient sample mass. Bromine non-detection indicates no measurable brominated flame-retardant carryover in this sample set and reduces the risk of brominated dioxin/furan formation during thermal processing. This conclusion is consistent with WD-XRF screening and with independent third-party combustion-IC cross-checks (Supplementary Section S1).

Chlorine (Cl) was detected in multiple samples (Table 8). For the measured set, Cl concentrations ranged from ~47 to 488 mg/kg (≈0.005–0.05 wt%), consistent with trace PVC/PVDC/adhesive contamination rather than bulk halogenated plastics. Sample P2 could not be analyzed for CIC because of insufficient material. Notably, the highest Cl level was observed in P1 (~488 mg/kg) despite its otherwise ‘clean’ industrial profile, underscoring that even well-sorted polyolefin streams can contain problematic trace chlorinated contaminants.

Iodine (I) was below the CIC reporting limit in all analyzed samples summarized in Table 8; iodine-containing plastics are uncommon in the studied waste categories.

From a recyclability standpoint, chlorine is the primary halogen of concern in these samples. Chlorine at the levels found (≈50–500 ppm; ~0.005–0.05 wt%) can have several detrimental effects. In mechanical recycling, even a fraction of a percent of PVC can ruin batches of polyolefins by causing discoloration and release of HCl at processing temperatures ~180–240 °C. In pyrolysis or incineration, chlorine can form HCl gas and catalyze the formation of dioxins and furans if not managed [78]. Indeed, studies show that just a few hundred ppm of chlorine in feedstock can yield oils with organochlorine levels that render them unsuitable for use without further refining [79,80,81]. Our data confirm the need for dechlorination steps or strict PVC removal before thermal recycling of these wastes. For example, P1, though mostly clean HDPE, should have that trace PVC removed (e.g., via density separation) before processing. The lack of Br suggests these wastes will not produce brominated dioxins under thermal treatment, and no special bromine scrubbing will be needed in pyrolysis flue gases. This is fortunate, as brominated flame retardants require more complex handling (their absence indicates our samples are primarily packaging, not electronic plastics).

In conclusion, the halogen analysis indicates that chlorine contamination is the foremost concern among halogens for this set of samples, with Cl detected at low-ppm to few-hundred-ppm levels in multiple streams (Table 8). Fluorine, bromine, and iodine were below reporting limits, simplifying the hazard profile (no detectable PFAS/BFR signatures in the analyzed set). Independent third-party combustion-IC cross-checks corroborate bromine non-detection and low chlorine levels for the tested materials (Supplementary Section S1). To safely recycle these materials, any chlorine-rich pieces (e.g., PVC fragments) should be removed or neutralized. Thermal processes must be equipped with acid-gas scrubbers and, where appropriate, catalytic baghouses to destroy any chlorinated organics formed. Mechanical recycling should avoid blending higher-Cl fractions into products that are not designed to tolerate chlorine. These findings underscore the need for stringent sorting (to eliminate PVC) and, where needed, dechlorination treatments for streams like P1 or P7–P9 if they are destined for pyrolysis.

#### 3.3.5. Elemental Signatures and Potential Sources in Plastic Waste Samples

Table 9 presents a focused overview of the principal inorganic elements detected across the plastic waste samples (P1–P9) and their likely sources. The retained entries are limited to elements supported by the elemental or halogen data reported in this manuscript. Common elements such as calcium, zinc, titanium, silicon, sodium, and potassium reflect widespread use of fillers, pigments, stabilizers, or process residues, while chlorine and lead point to contamination pathways that are particularly relevant to recycling-route selection and product safety. Understanding these elemental profiles helps identify contaminant sources and informs safe, tailored recycling or pretreatment strategies.

## 4. Recycling Recommendations and Practical Implications

Overall, the characterization reveals clear differences between samples, consistent with cleaner production scrap and with more heterogeneous consumer/commercial histories. Samples such as P1, P3, and P4 showed relatively clean single-polymer signatures, low residue, and limited oxidation/filler content, whereas P2, P5, P7, and P8 showed stronger evidence of fillers, oxidation, or mixed-stream contamination. Because sample-specific chain-of-custody information was unavailable, these distinctions should be interpreted as screening-based inferences rather than definitive provenance assignments.

From a practical standpoint, the full analytical suite employed in this study is research-grade and would not be applied to every incoming bale at a recycling facility. Among the techniques used, FTIR and DSC are rapid and low-cost (typically <US $10–30 per sample; turnaround < 15 min per scan), non-destructive or requiring only milligram quantities, and generate no hazardous waste. TGA/DTG adds moderate cost (~$30–60 per sample; ~2 h including temperature ramp and cooldown) and consumes only a few milligrams. These three front-line techniques are therefore well suited for routine quality-control screening. More specialized methods such as ICP-OES, WD-XRF, and CIC involve higher per-sample costs (~$50–150), require acid digestion or combustion (generating small volumes of chemical waste), and have longer turnaround times (~1–3 days including digestion). TG–MS is similarly a higher-tier method (~$80–120 per run) best reserved for confirmatory or diagnostic purposes. We therefore envision a tiered deployment model: Tier 1 (FTIR + DSC) for rapid polymer identification and subclass confirmation on all incoming streams; Tier 2 (TGA/DTG + proximate/ultimate analysis) for thermal stability and composition assessment on borderline or unknown feedstocks; and Tier 3 (ICP-OES, XRF, CIC, TG–MS) for targeted contaminant screening triggered by Tier 1/2 flags (e.g., anomalous residue, unexpected spectral bands, or regulatory thresholds). This staged approach minimizes unnecessary testing, limits analytical waste generation, and concentrates the more resource-intensive methods where they provide the greatest decision-support value. The cost estimates above are approximate and will vary with laboratory infrastructure, throughput, and local pricing; however, they illustrate that the front-line screening tools are economically viable for routine use, while the full suite is justified for initial characterization of new or suspect streams.

Regarding the environmental footprint of the analytical procedures themselves, the Tier 1 and Tier 2 methods (FTIR, DSC, TGA/DTG) are inherently low-impact: they consume only milligram-scale samples, use no solvents, and generate no hazardous waste beyond minute residual ash collected in inert crucibles. The Tier 3 methods carry a larger footprint: ICP-OES and CIC require acid digestion or combustion, consuming small volumes of mineral acids (HNO_3_, HCl; typically 10–20 mL per sample) and producing dilute aqueous waste that must be neutralized and disposed of as chemical effluent. WD-XRF is non-destructive and solvent-free, but does require pressed pellet preparation. TG–MS uses only inert carrier gas and produces negligible waste. Overall, a complete nine-sample campaign as performed here generates less than ~500 mL of dilute acid waste (from ICP-OES digestions) and less than 0.5 L of combustion rinse solution (from CIC), quantities that are routinely handled by standard laboratory waste management protocols and that are negligible compared with the environmental benefit of correctly routing tonnes of plastic waste away from landfill or uncontrolled incineration. The tiered approach further minimizes this footprint because the resource-intensive Tier 3 analyses are only triggered when Tier 1/2 screening flags an anomaly, avoiding unnecessary chemical consumption for clean, readily recyclable streams.

On a positive note, none of the samples showed elevated levels of hazardous additives, such as brominated flame retardants (BFRs), or other persistent organic pollutants. Bromine was undetected in all samples, indicating these wastes are primarily packaging-type materials rather than electronics or automotive shredder residue. This is encouraging because it reduces concern for brominated dioxin formation or the need for dedicated BFR removal processes. The dominant polymers across all samples are polyolefins (PE and PP), which is favorable from a recycling standpoint: when sufficiently free of contaminants, these polymers can be mechanically recycled or converted via pyrolysis into hydrocarbon products akin to refinery fuels. Prior studies of polyolefin pyrolysis oils report predominantly aliphatic hydrocarbons that are suitable as fuels or feedstocks after appropriate refining. One point to watch is the presence of olefins in pyrolysis-derived oils—high olefin content can cause gum formation and may require mild hydrogenation for stability [80]. This suggests that while chemical recycling of these samples is feasible, some downstream upgrading might be necessary for the liquid products.

Based on the specific contamination profiles and polymer compositions we determined, each waste plastic sample (or group of similar samples) requires a tailored recycling approach. We summarize our recommendations as follows:

P3, P4, P9 (mainly PP): These are relatively pure polypropylene wastes, making them suitable for closed-loop mechanical recycling into new PP products. P9 contains some filler and moisture, so minor preprocessing (drying and removing any non-PP attachments) is advised. Overall, its high PP purity is ideal for reprocessing. Maintaining separate PP recycling streams for these can yield high-quality recyclate.

P1, P2, P6 (cleaner PE/PP-rich streams or blends): These can be mechanically recycled if properly sorted and purified (especially P2, which is an HDPE-rich stream and a strong candidate for conventional recycling after contaminant removal). At the same time, they are also good candidates for chemical recycling (pyrolysis) because they have high volatile content and low intrinsic halogen burden. For P1, it is critical to remove any PVC fragments, given their detectable chlorine content; this can be done via density separation, as PVC is denser than HDPE. Once dechlorinated, P1’s mostly HDPE composition would be expected to yield a cleaner recyclate or oil.

P5, P7, P8 (filled or aged PE films): These are heavily contaminated with fillers (CaCO_3_) and have undergone aging (oxidation) in use, which makes their mechanical properties inferior. They are likely best suited for chemical recycling (pyrolysis) or, if not economically viable, energy recovery. Before pyrolysis, pre-washing to remove surface contaminants (dirt, salts) would improve process performance. During pyrolysis, their high ash content results in greater char production, so units should be equipped to handle solid residues and possibly utilize them (e.g., in cement). If these materials are ever reintroduced into new plastics, blending with virgin polymer or adding stabilizers will be necessary to compensate for their degraded properties.

Highly contaminated cases (any sample with excessive chlorine or toxic metals): When a batch is so contaminated that recycling is impractical or unsafe, controlled incineration with energy recovery becomes the option of last resort. This should be done in facilities with proper flue-gas cleaning to capture HCl, heavy metals, and any dioxins. For example, if a future batch resembles P7 or P9 and its detectable Pb or other trace metals are judged unacceptable for the intended product route, sending that material to a modern waste-to-energy plant with acid-gas scrubbers and dust filters may be more appropriate than attempting closed-loop reuse. Such disposal should remain a fallback after all recycling options have been exhausted.

Implementing these recommendations will help ensure that the recycling process for each waste stream is both efficient and environmentally responsible, maximizing material recovery while minimizing hazardous emissions. In practice, an optimal solution will likely involve a combination of strategies: advanced sorting and mechanical recycling for the clean fractions, and chemical or thermal treatment for the contaminated fractions. Our findings, when compared with prior studies, validate that these samples behave as expected for their type—e.g., post-consumer films inevitably contain fillers and require pyrolysis or incineration, whereas clean industrial scraps can be recycled into new products. Importantly, our data underscore the critical points that must be managed to make recycling safe (chlorine content, heavy metals, etc.). This knowledge helps develop an optimized recycling program for mixed post-consumer/commercial/industrial plastics, moving us closer to a more sustainable circular economy for plastics.

## 5. Conclusions

In this work, we established a comprehensive analytical framework to characterize mixed plastic waste feedstocks and identify their contaminants, directly linking those findings to appropriate recycling pathways. By applying a battery of advanced techniques (FTIR, DSC, TGA/TG–MS, ICP-OES, and CIC), we confirmed that all nine samples (P1–P9) are overwhelmingly composed of polyolefins (PE and PP), but with varying degrees of fillers and legacy contaminants. Clean, homogeneous samples (e.g., cleaner industrial scraps) can be more readily looped into mechanical recycling, whereas dirtier, more heterogeneous samples (especially consumer-like films with CaCO_3_ and Cl contamination) are better suited for chemical recycling or energy recovery with suitable emission controls. We provided concrete recycling recommendations for each sample group and demonstrated that even low levels of certain contaminants, such as ~0.005–0.05% chlorine (≈50–500 ppm) or detectable lead in selected streams, can influence processing choices.

Overall, our multi-technique approach proved effective at uncovering the “hidden” contamination profile of each waste stream—information vital for designing safe and efficient recycling processes. The results highlight the need for rigorous upstream sorting (to remove PVC and e-waste plastics) and, if necessary, pretreatment steps (washing, dehalogenation) for mixed wastes before recycling. They also show that with proper handling, the predominance of polyolefins in these wastes is a strength: these materials can be transformed into valuable recycled products or fuels, supporting circular economy goals. By systematically linking waste characterization to tailored recycling strategies, this study provides a model for how the plastics recycling industry can meet emerging regulatory standards and sustainability targets. Our findings echo broader calls for science-based solutions in tackling plastic pollution, demonstrating how detailed analytical data can directly inform policy and practice.

Practical deployment and resource considerations: While the full analytical suite is valuable for comprehensive, forensic-quality feedstock assessment, routine plant monitoring can be implemented as a tiered workflow. A rapid screening tier can rely on ATR-FTIR (polymer family), DSC (PE subclass discrimination, where needed), and WD-XRF for qualitative screening of halogens/metals. A confirmatory tier can be reserved for flagged streams and regulatory-relevant decisions, including CIC for total halogens and ICP-OES for trace metals. A deeper diagnostic tier (e.g., TG–MS and GPC) can be applied selectively to investigate degradation state, filler chemistry, and process-relevant behavior. This tiered approach minimizes consumables and analytical burden; the main wet-chemical waste streams are associated with acid digestion (ICP-OES) and, where applicable, solvent use, while FTIR/DSC/TGA/XRF are largely low-consumption methods.

In sum, this study delivers an in-depth feedstock profiling that diagnoses key challenges (e.g., chlorine, heavy metals, fillers, and degradation indicators) in mixed plastic waste and links them to actionable, fit-for-purpose recycling recommendations. The integrated methodology demonstrated here can be replicated and adapted to other waste streams, helping industry and regulators make data-driven decisions to achieve safer, more circular plastic life-cycles in line with emerging chemical-transparency expectations. A comprehensive list of acronyms used throughout this manuscript is provided in Table 10.

## Figures and Tables

**Table 1:** Thermogravimetric data of plastics.

Sample ID	T_0_ (°C)	T_f_ (°C)	T_max_ (°C)	ΔTG (%)
P1	365	502.2	475	99.99
P2	376	503.3	476	90.77
P2	611	673.3	658	2.32
P2	Total ΔTG Total ΔTG Total ΔTG			93.09
P3	353	485.5	458	98.23
P4	416	486.3	459	99.23
P5	392	519.9	477	91.23
P5	620	679.9	667	3.69
P5	Total ΔTG Total ΔTG Total ΔTG			94.92
P6	355	504.7	462	99.29
P7	417.9	520.4	482.9	94.89
P7			660.4	1.3
P7	Total ΔTG Total ΔTG Total ΔTG			96.19
P8	410.3	535.3	475.3	93.25
P8			687.8	3.12
P8	Total ΔTG Total ΔTG Total ΔTG			96.37
P9	376	503.3	460.7	95.6
P9			680.7	1.86
P9	Total ΔTG Total ΔTG Total ΔTG			97.46

**Table 2:** TG–MS evolved gas summary for selected plastic samples (P1–P6). Each sample’s dominant mass fragment ions are listed with their corresponding volatile species, and the interpretation relating to polymer identity and additives/fillers is provided.

Sample	Dominant *m*/*z* Fragments	Key Evolved Species (Assignment)	Interpretation (Polymer Type and Additives)
P1	28, 29, 41, 43	C_2_H_4_ (ethylene), C_2_H_6_ (ethane), C_3_H_6_ (propylene), C_3_H_8_ (propane)	– Pure PE (HDPE/LDPE) – Typical C_2_–C_3_ hydrocarbon gases – No filler detected (no CO_2_)
P2	18, 28, 29, 41, 43, 44	H_2_O (water), C_2_H_4_, C_2_H_6_, C_3_H_6_, C_3_H_8_, CO_2_ (carbon dioxide)	– HDPE with PE pyrolysis volatiles – CO_2_ release confirms CaCO_3_ filler – Elevated H_2_O from additives/aging (NIAS)
P3	28, 41, 43	C_2_H_4_, C_3_H_6_, C_3_H_8_	– Polypropylene (PP) – Dominated by C_3_ hydrocarbons (propylene/propane) – No filler or moisture
P4	28, 41, 43	C_2_H_4_, C_3_H_6_, C_3_H_8_	– Polypropylene (similar to P3) – Mainly C_3_ pyrolysis products – No filler present
P5	28, 29, 41, 43, 44	C_2_H_4_, C_2_H_6_, C_3_H_6_, C_3_H_8_, CO_2_	– LDPE (with minor HDPE) – Typical PE volatiles – CO_2_ from significant CaCO_3_ filler
P6	28, 29, 41, 43	C_2_H_4_, C_2_H_6_, C_3_H_6_, C_3_H_8_	– Mixed PE/PP blend – Both C_2_ and C_3_ fragments – No notable CO_2_ (minimal filler)

**Table 3:** GPC results for selected plastic samples (P1–P6).

Sample	Mp	Mn	Mw	Mz
P1	147,040	44,959	238,891	683,064
P2	137,869	44,937	228,606	596,549
P3	156,596	76,526	197,794	365,870
P4	188,593	56,685	250,303	575,682
P5	99,747	43,309	235,918	899,948
P6	122,743	48,623	170,115	375,282

**Table 4:** Integrated multi-technique summary of polymer identity, thermal behavior, and key contamination indicators for samples P1–P9.

Sample	Identified Polymer(s)	Spectral Signatures (FTIR)	Thermal Behavior (DSC and TGA/TG–MS)	Additives/Contamination Evidence	Likely Origin/Stream Character
P1	Poly(ethylene)—mixture of LDPE and HDPE	– CH_2_ rocking/bending modes – C–H stretches confirm PE – Raman confirms PE fingerprint	– DSC: peaks ~110 °C (LDPE), ~130 °C (HDPE) – TGA: ~475 °C, ~99% loss – TG–MS: C_1_–C_3_ hydrocarbons only (pure, no fillers)	– No additives detected – No carbonyl bands – <0.5% ash (essentially pure)	– Likely cleaner production scrap
P2	Poly(ethylene)—high-density (HDPE)	– CH_2_ modes (PE base polymer) – Weak C=C/O–H bands (mild oxidation) – Trace aromatic impurities; Raman confirms PE	– DSC: ~130 °C (HDPE) – TGA: 2-stage (~476 °C, ~700 °C) – TG–MS: C_1_–C_3_ hydrocarbons + notable CO_2_ release (>650 °C) confirms CaCO_3_ filler decomposition; ~6.6% residual ash	– CaCO_3_ filler (6–7% ash, CO_2_ evolution) – Mild oxidation (C=C, O–H bands) – Evidence of weathering/aging	– Likely filled/aged consumer-like HDPE stream
P3	Poly(propylene) (PP)	– CH_2_/CH_3_ vibrations (isotactic PP) – Raman confirms PP fingerprint	– DSC: ~160 °C (PP) – TGA: single-stage ~460 °C, ~99% loss – TG–MS: C_3_ hydrocarbons (pure PP)	– No additives – No carbonyl bands – ~1% ash (no filler)	– Likely cleaner production scrap
P4	Poly(propylene) (PP)	– CH_3_/CH_2_ bands (PP polymer) – Raman weak (fluorescence) but consistent with PP	– DSC: ~160 °C (PP) – TGA: single-stage ~458–460 °C, ~99% loss – TG–MS: C_3_ hydrocarbons only (pure)	– No additives – No oxidation bands – ~0.9% ash (no filler)	– Likely cleaner production scrap
P5	Poly(ethylene)—high-density (HDPE)	– CH_2_ bands (HDPE base) – Broad C=O (~1710 cm^−1^) indicates oxidized PE – Raman confirms PE polymer backbone (matches PE standard)	– DSC: ~125–130 °C (HDPE) – TGA: multi-stage (~477 °C, ~700 °C) – TG–MS: C_1_–C_3_ hydrocarbon volatiles + strong CO_2_ evolution (700 °C peak) confirms filler decomposition; ~10% residue	– CaCO_3_ filler (10.2% ash, CO_2_ evolution) – Significant oxidation (carbonyl bands) – Evidence of weathering; possibly stabilizers/additives present	– Likely filled/aged consumer-like HDPE stream
P6	Polyolefin blend—LDPE, HDPE and PP	– CH_2_/CH_3_ bands (PE/PP blend) – Weak C=O (slight oxidation) – Raman inconclusive	– DSC: ~110 °C (LDPE), ~130 °C (HDPE), ~165 °C (PP) – TGA: multi-step, ~460 °C (PP), ~470–480 °C (PE), ~98% loss – TG–MS: C_2_–C_3_ fragments from both PE and PP; no high-T gas release (low ash ~2%)	– Minor oxidation (trace carbonyl) – <2% ash (no substantial filler) – No significant additives	– Likely mixed consumer/commercial polyolefin stream
P7	Poly(ethylene)—low and high density (LDPE and HDPE)	– CH_2_ rocking/stretching (PE) – C=C band (unsaturation/oxidation) – Raman not measured	– DSC: ~110 °C (LDPE), ~130 °C (HDPE) – TGA: 2-step, ~475 °C + minor to ~750 °C – TG–MS: PE hydrocarbons, minor CO_2_ (CaCO_3_); ~3% residue	– CaCO_3_ filler (~2.8% ash, 700 °C peak) – Oxidation (C=C, O–H bands) – Low-level additives	– Likely heterogeneous consumer/commercial PE stream
P8	Poly(ethylene)—high-density (HDPE)	– CH_2_ vibrations (PE) – Faint C=C (oxidative unsaturation) – Raman not measured	– DSC: ~125 °C (HDPE) – TGA: near single-step ~470 °C, minor ~700 °C; ~4–5% residue – TG–MS: C_2_–C_3_ hydrocarbon + minor CO_2_	– CaCO_3_ filler (~4.7% ash, CO_2_ release) – Mild oxidation (aging/additive breakdown) – Moderate inorganic signature	– Likely heterogeneous consumer/commercial HDPE stream
P9	Polyolefin mix—HDPE and PP	– CH_2_/CH_3_ bands (PE/PP mixture) – C=C (unsaturation/additive) – Raman not measured	– DSC: ~130 °C (HDPE), ~165 °C (PP) – TGA: multi-stage, ~460–480 °C, subtle secondary; ~3% residue – TG–MS: not run; expected PE/PP gases (low filler)	– Minor inorganic (~3.4% ash, pigment/filler) – Slight oxidation (C=C bands) – No significant additives	– Likely commercial mixed polyolefin stream

**Table 5:** Proximate analysis of plastic waste samples P1–P9 (wt%, dry basis).

Plastics	Proximate Analysis (wt%)			
Plastics	Moisture	Volatile matter	Fixed Carbon	Ash
**P1**	0.07	99.41	0.02	0.50
**P2**	0.12	93.29	0.01	6.58
**P3**	0.05	96.80	0.93	2.22
**P4**	0.07	98.92	0.90	0.11
**P5**	0.07	89.38	0.34	10.21
**P6**	0.13	97.00	0.99	1.88
**P7**	0.06	96.10	1.01	2.83
**P8**	0.02	92.62	2.70	4.66
**P9**	0.20	95.58	0.95	3.37

**Table 6:** Ultimate (CHNSO) analysis of plastic waste samples P1–P9 (wt%, dry basis). ND: not detected (detection limit for N is 0.1%).

Plastics	Elemental Composition (wt%)				
Sample	C	H	N	O	Total
**P1**	85.90	14.03	ND ^a^	1.23	101.16
**P2**	79.90	12.68	ND	1.46	94.05
**P3**	84.20	13.43	ND	0.92	98.55
**P4**	86.04	13.66	ND	0.61	100.31
**P5**	84.03	13.15	ND	1.37	98.55
**P6**	84.71	13.36	ND	0.82	98.88
**P7**	82.92	14.03	0.14	1.52	98.60
**P8**	79.80	13.23	0.14	2.85	96.02
**P9**	82.57	13.94	0.11	2.00	98.63

**Table 7:** ICP-OES results of waste plastic samples.

Element (ppm)	Al	As	Ba	Ca	Cd	Fe	K	Mg	Na	P
P1	54.05	ND	ND	27.43	ND	ND	ND	ND	9.35	20.99
P2	1155.80	ND	ND	26,900.00	ND	28.28	4993.74	2718.60	9698.56	121.36
P3	76.17	ND	ND	5362.95	ND	14.67	ND	25.57	44.19	38.34
P4	37.81	ND	ND	881.50	ND	25.15	ND	31.06	85.88	40.51
P5	213.93	ND	ND	15,362.08	ND	14.52	ND	57.62	19.93	90.88
P6	185.45	ND	ND	152.80	ND	ND	ND	20.44	16.51	26.76
P7	216.80	ND	22.51	15,401.03	ND	74.28	21.15	404.86	106.25	34.69
P8	68.92	ND	ND	35,608.29	ND	18.53	ND	75.49	24.40	70.97
P9	216.86	ND	50.58	20,896.63	ND	335.78	15.58	445.74	86.72	45.71
Element (ppm)	Pb	S	Sb	Si	Sn	Sr	Te	Ti	Zn	Sum./%
P1	ND	ND	ND	222.47	ND	ND	ND	ND	ND	0.03
P2	ND	332.83	ND	827.45	ND	ND	ND	451.88	53.68	4.73
P3	ND	91.17	ND	43.24	ND	ND	ND	208.8	35.72	0.59
P4	ND	16.73	ND	74.01	ND	ND	ND	33.15	6.8	0.12
P5	ND	279.02	ND	117.46	ND	ND	ND	277.05	30.65	1.65
P6	ND	12.5	ND	94.02	ND	ND	ND	232.33	24.7	0.08
P7	20.56	268.74	ND	733.47	ND	99.78	ND	1664.25	153.23	1.92
P8	ND	451.26	ND	127.88	ND	12.18	ND	1124.93	52.51	3.76
P9	9.98	291.14	ND	367.09	ND	10.88	ND	1828.17	28.91	2.46

**Table 8:** Halogen concentrations in the waste plastic samples.

Sample	F (ppm)	Cl (ppm)	Br (ppm)	I (ppm)
P1	ND.	488.14	ND.	ND.
P2 #	N.A.	N.A.	N.A.	N.A.
P3	ND.	120.94	ND.	ND.
P4	ND.	189.52	ND.	ND.
P5	ND.	67.57	ND.	ND.
P6	ND.	47.30	ND.	ND.
P7	ND.	179.36	ND.	ND.
P8	ND.	116.94	ND.	ND.
P9	ND.	62.41	ND.	ND.

Detection limit: 1 ppm; ND: not detected; # P2 sample mass was insufficient for CIC; no result available (NA).

**Table 9:** Detected inorganic elements and their likely sources in plastic waste samples P1–P9. This information helps trace material provenance and informs pretreatment and recycling strategies to manage legacy contaminants and improve process safety and product quality.

Element	Detected in Samples	Source Description
Al (Aluminum)	P1–P9	– Packaging components, mineral residues, additive packages – Metallized films or process contamination in recycled streams
Ba (Barium)	P7, P9	– Pigments, fillers, colorant systems – White pigments, pigment extenders in consumer/commercial plastics
Ca (Calcium)	P1–P9	– CaCO_3_ filler (stiffness, cost reduction) – Ca-based stabilizers and processing aids
Cl (Chlorine)	P1, P3–P9	– PVC/PVDC fragments, chlorinated adhesives/additives – Minor contamination, not bulk halogenated plastics
Fe (Iron)	P2–P5, P7–P9	– Iron-oxide pigments – Wear debris from shredding/extrusion equipment – Mixed waste handling contamination
K (Potassium)	P2, P7, P9	– Salts and processing residues – Fertilizer contamination, prior-use residues
Mg (Magnesium)	P2–P9	– Mg-containing fillers and lubricants – Stabilizers (magnesium stearate, magnesium hydroxide)
Na (Sodium)	P1–P9	– Salt residues, washing/process chemicals – Sodium-containing additives/contaminants
P (Phosphorus)	P1–P9	– Organophosphate flame retardants – Plasticizers and antioxidants
Pb (Lead)	P7, P9	– Low ppm levels from legacy pigments – Mixed waste handling, Pb-component contact – Caution for sensitive applications
S (Sulfur)	P2–P9	– Pigments, sulfur-containing stabilizers – Antioxidants, prior-use/contamination residues
Si (Silicon)	P1–P9	– Silica/silicate/silicone fillers – Mechanical property/processability modification
Sr (Strontium)	P7–P9	– Pigment/filler systems – Contamination from mixed commercial/consumer waste
Ti (Titanium)	P2–P9	– TiO_2_ pigment – Opacity, whiteness, UV protection
Zn (Zinc)	P2–P9	– Zinc stearate, ZnO, pigment systems – Stabilizer packages in plastic processing

**Table 10:** List of acronyms used in the manuscript.

List of Acronyms	
Acronym	Full Form
ABS	Acrylonitrile Butadiene Styrene
ATR	Attenuated Total Reflectance
BFR	Brominated Flame Retardants
CERP	Clean Energy Research Platform
CHNSO	Carbon, Hydrogen, Nitrogen, Sulfur, and Oxygen (Ultimate Elemental Analysis)
CIC	Combustion Ion Chromatography
DSC	Differential Scanning Calorimetry
DTG	Derivative Thermogravimetry
EU	European Union
FTIR	Fourier Transform Infrared Spectroscopy
GC-MS	Gas Chromatography–Mass Spectrometry
GC×GC–TOF–MS	Comprehensive Two-Dimensional Gas Chromatography with Time-of-Flight Mass Spectrometry
GPC	Gel Permeation Chromatography
HDPE	High-Density Polyethylene
ICP-OES	Inductively Coupled Plasma–Optical Emission Spectroscopy
LDPE	Low-Density Polyethylene
MSW	Municipal Solid Waste
NIAS	Non-Intentionally Added Substances
PSE	Physical Science and Engineering Division (KAUST)
PET	Polyethylene Terephthalate
PMMA	Polymethyl Methacrylate
POPs	Persistent Organic Pollutants
PP	Polypropylene
PS	Polystyrene
PVC	Polyvinyl Chloride
Py-GC/MS	Pyrolysis Gas Chromatography–Mass Spectrometry
RoHS	Restriction of Hazardous Substances
SCW	Supercritical Water
SDG	Sustainable Development Goals
SVOCs	Semi-Volatile Organic Compounds
TGA	Thermogravimetric Analysis
TG–MS	Thermogravimetry–Mass Spectrometry
UNEP	United Nations Environment Programme
VOCs	Volatile Organic Compounds
WD-XRF	Wavelength Dispersive X-ray Fluorescence
WEEE	Waste Electrical and Electronic Equipment
XRF	X-ray Fluorescence

## Data Availability

The original contributions presented in this study are included in the article/Supplementary Material. Further inquiries can be directed to the corresponding author.

## Supplementary Materials

The following supporting information can be downloaded at: https://www.mdpi.com/article/10.3390/polym18101256/s1. Table S1: Representative overview of key studies (2015–2026) on mixed plastic waste characterization; Table S2: FTIR peaks identified for plastic samples; Table S3: Third-party combustion-IC halogen results used for cross-validation; Table S4: Apparent carbonyl index derived from ATR-FTIR transmittance spectra; Table S5: Ion fragments from TG–MS analysis of plastic samples; Table S6: Main ion fragments corresponding to possible compounds; Figure S1: Representative Raman spectra of common thermoplastics (PE, PP, PET, PMMA, PVC); Figure S2: Raman spectra of selected plastic waste samples (P1–P6); Figure S3: TG–MS profiles of mixed plastic waste samples (P1–P6); Figure S4: Thermogravimetric proximate-analysis profiles of samples P7 (LDPE), P8 (HDPE), and P9 (PP); Section S1: Halogen analysis method selection and inter-laboratory validation; Section S2: Apparent carbonyl index from ATR-FTIR spectra.
