# Supplementary material for: A Tiered Multi-Technique Decision-Support Framework for Contaminant Screening and Recycling-Route Assignment of Mixed Plastic Waste
Source: Polymers (Basel). 2026 May 21;18(10):1256. doi: 10.3390/polym18101256 (PMC13210819; doi:10.3390/polym18101256)
Supplement: Supplementary file 1 [file polymers-18-01256-s001.zip › polymers-4185254-supplementary.pdf]

Supplementary Material

Supplementary Information: A Tiered Multi-Technique Decision-Support Framework for Contaminant Screening and Recycling-Route Assignment of Mixed Plastic Waste

Aiping Chen [1,†], Saumitra Saxena [1,*], Vasilios G. Samaras [2] and Bassam Dally [1]

[1] Clean Energy Research Platform, Physical Science and Engineering Division (PSE), King Abdullah University of Science and Technology, Thuwal 23955-6900, Saudi Arabia; aiping@mail.ust.edu.cn (A.C.); bassam.dally@kaust.edu.sa (B.D.)
[2] Analytical Chemistry Core Lab, King Abdullah University of Science and Technology, Thuwal 23955-6900, Saudi Arabia; vasileios.samaras@kaust.edu.sa
* Correspondence: saumitra.saxena@kaust.edu.sa
† Current address: School of Materials Science and Engineering, Suzhou University of Science and Technology, Suzhou 215009, China

Table S1: Representative overview of key studies (2015–2026) on mixed plastic waste characterization from post-consumer, commercial, and industrial sources. Grouped by waste type and analytical focus, the table highlights methods such as FTIR, Raman, TGA, Py-GC/MS, XRF, and machine-learning-assisted spectroscopy used to assess polymer types, contaminants, and recyclability. It captures the scope (laboratory, pilot, model) and the relevance to recycling strategies, including sorting challenges, representative sampling, contaminant screening, and chemical recovery options.

	Study (Authors & Year)	Waste Stream Type	Analytical Focus	Analytical Scope & Scale	Recycling Relevance
1	Dahlbo et al., 2018[1]<br><br>– Evaluated the recycling potential of post-consumer plastic packaging in Finland. Composition analysis (incl. ~17–20% multilayer packaging) was used to model scenarios.<br>Considered mechanical vs. chemical recycling yields and environmental/economic performance. *Scale:* national data/modeling (field sampling + material flow analysis). *Comparison:* mechanical sorting/recycling vs. feedstock (pyrolysis) and incineration scenarios. *Advanced aspects:* scenario-based optimization of a recycling chain for maximum material recovery. *Real-world:* informs Finnish policy (EPR targets) and system design for packaging waste.	Post-consumer (MSW packaging)	Process modeling; Polymer ID (composition)	Scope: National-level analysis of sorted packaging waste by polymer type and product type; incorporated impurity and multilayer fractions into recycling models. No lab detection limits (uses waste statistics and composition audits). Sensitivity: Evaluates system-level recycling rates rather than chemical detection. Scale: Study uses real waste composition data and simulation (no lab analytical threshold). Method comparisons: Compared recycling routes – mechanical vs. pyrolysis vs. energy recovery – in terms of achievable recycling rate	Mechanical & Chemical; Policy Design (optimizing recycling vs. incineration)
2	Faraca & Astrup, 2019[2]<br><br>– Characterized plastics from recycling centers (post-consumer bulky waste): separate streams of hard plastics, films, and PVC from three sites. Analyzed by product type, polymer, "quality" (legislative category), and impurities. *Scale:* pilot/field study (bulk sampling ~100–200 kg). *Comparison:* "High-quality" (e.g. food-grade) vs "Low-quality" plastics and their recyclability. Found 28% impurities (metal, multi-material, dark color) overall and ~75% of items were low-quality applications. Recycling potential:	Post-consumer (drop-off center hard plastics, films, PVC)	Polymer ID; Contaminant analysis; Process modeling	Scope: Detailed composition by polymer (PE, PP, PS, PVC, etc.), product use (e.g. packaging, toys), and contaminants (non-plastics, multi-layer items). Identified colored or brominated pieces via visual/XRF. Sensitivity: Quantified impurities by weight (~28% of stream) – sufficient to calculate material flow impact. Scale: Field sampling of municipal bulky waste, lab sorting/ID, plus material-flow modeling for recycling scenarios. Method comparisons: Used mass balance modeling to compare idealized recycling of "high- vs low-quality" fractions (finding high-quality plastics are 12–35% more recyclable than low-quality).	Mechanical; Policy/Facility Design (improving sorting efficiency)

	Study (Authors & Year)	Waste Stream Type	Analytical Focus	Analytical Scope & Scale	Recycling Relevance
	52% for hard plastics, 59% for films, 79% for PVC when accounting for impurities. *Real-world:* highlights design improvements for sorting facilities and the need to remove contaminants to boost recycling.				
3	Roosen et al., 2020[3]<br><br>– In-depth composition analysis of mixed plastic packaging waste. Collected 102 packaging items from a Belgian sorting plant; separated each into components (bottles, caps, labels) for individual analysis. Applied multi-technique analytics: FTIR and DSC for polymer identification, polarized optical microscopy for structure, and elemental analysis (CHNSO, ICP-OES for metals, ion chromatography for halogens). *Scale:* laboratory (100+ individual samples, 242 sub-samples). Found waste streams with up to 9 different polymers and multiple additives; common metals (Ca, Al, Zn, Fe) and halogens (Cl, F) detected at 1–3000 ppm levels. *Comparison:* demonstrated how such heterogeneity and contaminant load impede closed-loop recycling, requiring advanced pretreatment. *Real-world:* provides a benchmark for packaging recyclers on contamination profiles and the need for decontamination before mechanical recycling or pyrolysis.	Post-consumer (household packaging)	Polymer ID; Additive/Contaminant analysis; Thermal analysis (DSC)	Scope: Comprehensive untargeted analysis of polymer composition and elemental contaminants in packaging waste. High analytical sensitivity – detected trace elements down to ~1 ppm. Scale: Laboratory characterization of sorted packaging components. Method comparisons: Integrated multiple analytical methods for cross-validation (e.g. FTIR vs. DSC for polymer ID) rather than comparing alternatives; highlighted the complementarity of chemical analysis techniques. Advanced techniques: Used ICP-OES and IC to quantify metals/halogens and DSC for thermal properties, providing a robust contaminant profile for each polymer type.	Mechanical & Chemical (pyrolysis) – underscores limits of closed-loop mechanical recycling with impure streams and supports chemical recycling for contaminated mixes.

	Study (Authors & Year)	Waste Stream Type	Analytical Focus	Analytical Scope & Scale	Recycling Relevance
4	Eriksen et al., 2018[4, 5]<br><br>– Investigated metal contamination in recycled plastics. Analyzed 82 samples of PET, PE, PP, PS from various origins: household waste items, recyclate pellets (household & industrial), and virgin resin. Used acid digestion + ICP-OES to quantify 15 metals (Al, Cd, Cr, Pb, Sb, etc.) in each sample. Separated samples by prior use (food-contact vs non-food) and tested a washing pre-treatment on waste plastics. *Scale:* lab analysis of real recyclate and waste plastic batches. *Findings:* Recycled plastics from household waste had systematically higher metal levels than industrial scrap or virgin plastic, though all measured concentrations were below current legal limits. Washing reduced surface dirt but many metal contaminants remained embedded. Statistical comparisons confirmed source (household vs industrial) significantly influences metal content. *Real-world:* shows that cumulative recycling may elevate trace metals in plastics, potentially affecting long-term quality and regulatory compliance. Supports the need for contaminant removal technologies or application of these materials in less sensitive products.	Post-consumer vs. Post-industrial (comparative)	Additive/Contaminant analysis (metals); Material quality assessment	Scope: Broader review context collating data on chemical additives present across plastic waste streams and their detection methods. Sensitivity: discusses analytical detection limits for migratable additives and cites studies on migration thresholds. Scale: literature review including both laboratory studies and real-world observations. Method comparisons: summarizes advantages of different analytical approaches reported in the literature and compares additive behavior in mechanical vs. chemical recycling.	Mechanical recycling – quality control (supports separating streams for high-end vs low-end uses); Policy (food safety standards for recycled plastics).
5	Hahladakis et al., 2018[6]<br><br>– Comprehensive review of additives in plastics and their fate	Multiple (Post-consumer	Additive/Contaminant analysis; (Review of migration & fate)		Mechanical & Chemical; Policy Design (hazardous substance restrictions, design for recyclability)

	Study (Authors & Year)	Waste Stream Type	Analytical Focus	Analytical Scope & Scale	Recycling Relevance
	during use and end-of-life. Covered common additives (plasticizers, flame retardants, stabilizers, pigments, etc.) and highlighted their potential to migrate, leach, or form toxic degradation products. Summarized analytical techniques for detecting these additives and noted knowledge gaps in mixtures. *Scale:* literature synthesis (no new experiments). *Advanced/emerging points:* Raised awareness of "legacy" additives in recycled plastics (e.g. brominated flame retardants from WEEE, heavy-metal stabilizers in legacy PVC) and challenges they pose to recycling. Discussed regulatory frameworks (REACH, etc.) and the need for better screening in recycling operations. *Real-world:* Highly policy-relevant – informs safety regulations and design-for-recycling to phase out or monitor hazardous additives in recycling streams.	packaging, WEEE plastics, etc.)			
6	Vijayan et al., 2021[7]<br><br>– Characterization of Automotive Shredder Residue (ASR) for pyrolysis. Investigated pre-treatment and thermal conversion of ASR (the mixed plastic/rubber "fluff" from end-of-life vehicles). Developed a process to homogenize ASR into a feedable powder. Conducted thermogravimetric analysis (TGA) under various heating rates and particle sizes to derive pyrolysis kinetics, and used DRIFTS (infrared spectroscopy of off-gases[8]) to identify	Post-consumer (Automotive Shredder Residue)	Thermal behavior (TGA kinetics); Polymer/volatile identification	Scope: Physical and chemical characterization of a highly mixed waste (plastics + foam + rubber). Focused on thermal decomposition profiles rather than detailed chemistry of each component. Sensitivity: High – TGA detects weight loss steps for fractions as small as <1% of sample; DRIFTS identifies evolved gases qualitatively (ppm-level detection of functional groups). Scale: Lab-scale simulation of pyrolysis (gram-scale samples). Method comparisons: Explored effect of particle size vs. heating rate on kinetic parameters; combined thermal analysis with spectral gas analysis for a fuller picture. Advanced: Introduced a novel pretreatment (shredding/fractionation) to improve processability of ASR.	Chemical (Pyrolysis); Energy Recovery (fuel production)

	Study (Authors & Year)	Waste Stream Type	Analytical Focus	Analytical Scope & Scale	Recycling Relevance
	volatiles released during thermal degradation. *Scale:* laboratory (bench-scale TGA/FTIR, particle size control). *Findings:* ASR shows multi-stage decomposition due to its heterogeneous polymer mix; optimized heating rates were proposed for efficient pyrolysis. Chlorinated and brominated compounds in off-gas were tracked via IR, informing emission control needs. *Real-world:* provides engineering data for scaling up ASR pyrolysis (supports achieving the 95% ELV recovery target by turning the plastic fraction into fuel/chemicals).				
7	Dzoh Fonkou et al., 2025[9]<br><br>– Review of analytical methods for in-depth assessment of recycled plastics, covering spectroscopic, thermal, elemental, and morphological techniques used to evaluate recycled-polymer quality and contamination.	Multiple recycled plastic streams	Integrated analytical methods; polymer/additive/contaminant characterization	Scope: review of FTIR, Raman, XRF, ICP, XRPD, DSC, SEM, and related methods for chemical, structural, morphological, and physical characterization of recycled plastics. Scale: literature review. Method comparisons: emphasizes complementarity of methods for quality assessment and contamination screening.	Mechanical & Chemical; strong support for multi-technique quality-control workflows in recycled plastics.
8	Amin et al., 2025[10]<br><br>– Comparative study of analytical techniques for identifying post-consumer plastic waste, highlighting the value of solid-state 13C NMR alongside FTIR and Raman for quantification of difficult polyolefin mixtures.	Post-consumer plastic waste / mixed waste samples	Comparative analytical methods for polymer identification and quantification	Scope: experimental comparison of FTIR, Raman, and solid-state 13C NMR for identifying common post-consumer plastics and quantifying mixed polyolefin waste. Scale: laboratory study. Method comparisons: demonstrates strengths and limitations of each method for mixed-stream analysis.	Mechanical recycling – feedstock ID and quantification; relevant for sorting, compositional QA, and mixed-polyolefin analysis.

	Study (Authors & Year)	Waste Stream Type	Analytical Focus	Analytical Scope & Scale	Recycling Relevance
9	Moreira et al., 2025[11]<br><br>– Demonstrated Raman spectroscopy for identifying and classifying postconsumer packaging plastics, including label/dye effects, with high classification accuracy for recycling codes 1–6.	Post-consumer packaging plastics	Raman spectroscopy for polymer identification and sorting	Scope: experimental Raman study of postconsumer packaging plastics with classification modeling. Scale: laboratory / applied sorting study. Method comparisons: shows Raman capability for identifying packaging plastics and handling dye/label signals relevant to sorting stations.	Mechanical recycling – improved sorting efficiency and reduced contamination in packaging-plastic streams.
10	Akbari Lakeh et al., 2025[12]<br><br>– Introduced a sampling-theory and thermal-analysis framework for determining representative sample size and estimating cross-contamination in plastic recyclate batches.	Industrial recyclate batches / mixed recyclate streams	Representative sampling; cross-contamination analysis; thermal-analysis-based QC	Scope: combines sampling theory with MADSCAN, a scale-free thermal analysis method, to estimate cross-contamination and define required sample size for heterogeneous recyclate batches. Scale: laboratory / industrial QC relevance. Method comparisons: addresses analytical vs. sampling error in recyclate characterization.	Mechanical recycling – highly relevant for defensible QC, batch representativeness, and contamination control.
11	Sorino et al., 2025[13]<br><br>– Bench-scale pyrolysis study of real contaminated mixed plastic waste from a pre-sorting waste stream, examining the influence of polymer composition, temperature, and residence time on yields and contaminant burden.	Real contaminated mixed plastic waste	Mixed-waste pyrolysis; contaminant analysis; process-parameter effects	Scope: semi-batch pyrolysis of contaminated real-world mixed plastic waste containing mainly LDPE, PP, and HDPE. Scale: bench-scale reactor study. Method comparisons: relates feed composition, temperature, and residence time to hydrocarbon yields and contaminant behavior.	Chemical recycling – directly relevant to realistic mixed-waste pyrolysis and downstream product-quality constraints.
12	Zhou et al., 2026[14]<br><br>– Developed ATR-FTIR plus machine-learning models for accurate identification of mixed and contaminated plastic waste, including dark/black plastics and oil-contaminated plastics without pre-cleaning.	Mixed and contaminated plastic waste	ATR-FTIR with machine learning for direct waste-plastic characterization	Scope: establishes two ATR-FTIR/ML models, including one for 10 common plastic types and another for oil-contaminated plastics using ICA-based spectral unmixing. Scale: laboratory / automated-sorting study. Method comparisons: addresses difficult streams such as dark plastics and contaminated plastics that challenge conventional sorting.	Mechanical recycling – strong relevance for advanced sorting automation, contaminated-waste characterization, and industrial screening.

Table S2: FTIR peaks identified for plastic samples.

Polymer / feature	Key FTIR bands (cm^{-1})	Assignment / interpretation	Samples (examples)
Polyethylene (PE)	2914–2847; 1464; ~720	C–H stretching (CH_2), CH_2 bending, CH_2 rocking	P1, P2, P5, P7–P9 (PE-rich)
Polypropylene (PP)	2917–2841; 1456; 1375; ~841	C–H stretching; CH_2/CH_3 bending; tertiary-carbon backbone band	P3, P4 (PP-rich); blends in P6/P9
Carbonyl/oxidation indicator	~1710–1740	C=O stretching (oxidation/weathering or oxygenated additives)	Observed qualitatively in oxidized/aged fractions (see main text)
Carbonate filler ($CaCO_3$)	~1410–1470; ~874; ~713	CO_3^{2-} vibrations (often overlaps with polymer CH_2 band)	P2, P5 and other filled streams
Aromatic/PS-like indicator (if present)	~1600; ~1490; ~700	Aromatic C=C stretching and out-of-plane C–H	Not dominant in P1–P9 set (screening)
PVC-like indicator (if present)	~600–700; 1250–1350	C–Cl stretching region (often weak in mixtures)	Not dominant; trace Cl assessed quantitatively by CIC (main text Table 8)

Section S1. Halogen analysis: method selection and inter-laboratory cross-validation

Halogens (F, Cl, Br, I) were assessed using multiple methods to ensure reliable quantification for heterogeneous plastic waste. WD-XRF was used as a rapid, non-destructive screening tool to flag potential halogen presence and guide subsampling; however, because of surface heterogeneity and matrix/calibration sensitivity at low ppm levels, quantitative reporting in this study is based on combustion ion chromatography (CIC) measurements performed at KAUST (main manuscript, Table 8).

To document detection thresholds and provide independent inter-laboratory validation, selected materials were additionally analyzed by two third-party laboratories using oxidative combustion followed by ion chromatography (combustion-IC). Centre Testing International (CTI, Ningbo) reports testing by IC referencing EN 14582:2016 with a stated method detection limit (MDL) of 10 mg/kg for each halogen; for an LDPE sample CTI measured Cl = 184 mg/kg and reported F/Br/I as not detected (<10 mg/kg), while HDPE and PP were reported as not detected (<10 mg/kg) for all halogens.

Beijing ZKGX Research Institute performed combustion-IC testing on selected waste samples and reports F/Br/I as not detected at <1 mg/kg, with chlorine detected in the low-ppm to few-hundred-ppm range for the tested subset (Table S5).

Overall, the third-party results corroborate two key conclusions used in the manuscript: (i) bromine is below detection limits in the analyzed materials (≤10 mg/kg in CTI testing and <1 mg/kg in ZKGX testing), and (ii) chlorine occurs at low levels consistent with trace chlorinated contaminants (e.g., minor PVC/PVDC/adhesives) rather than bulk halogenated plastics.

Table S3: Third-party combustion-IC halogen results used for cross-validation (reported in mg/kg; ND = not detected).

Laboratory / Source	Method / Standard	Sample	Reporting limit	F (mg/kg)	Cl (mg/kg)	Br (mg/kg)
CTI (Ningbo)	Combustion-IC; EN 14582:2016	LDPE (reference)	MDL 10 mg/kg	ND (<10)	184	ND (<10)
CTI (Ningbo)	Combustion-IC; EN 14582:2016	HDPE (reference)	MDL 10 mg/kg	ND (<10)	ND (<10)	ND (<10)
CTI (Ningbo)	Combustion-IC; EN 14582:2016	PP (reference)	MDL 10 mg/kg	ND (<10)	ND (<10)	ND (<10)
Beijing ZKGX (Zhongxi)	Combustion-IC (reporting: ND <1 mg/kg for F/Br/I)	P1	F/Br/I: <1 mg/kg	ND (<1)	488.14	ND (<1)
Beijing ZKGX (Zhongxi)	Combustion-IC (reporting: ND <1 mg/kg for F/Br/I)	P2	F/Br/I: <1 mg/kg	ND (<1)	Not analyzed (insufficient mass)	ND (<1)
Beijing ZKGX (Zhongxi)	Combustion-IC (reporting: ND <1 mg/kg for F/Br/I)	P3	F/Br/I: <1 mg/kg	ND (<1)	120.94	ND (<1)
Beijing ZKGX (Zhongxi)	Combustion-IC (reporting: ND <1 mg/kg for F/Br/I)	P4	F/Br/I: <1 mg/kg	ND (<1)	189.52	ND (<1)
Beijing ZKGX (Zhongxi)	Combustion-IC (reporting: ND <1 mg/kg for F/Br/I)	P5	F/Br/I: <1 mg/kg	ND (<1)	67.57	ND (<1)
Beijing ZKGX (Zhongxi)	Combustion-IC (reporting: ND <1 mg/kg for F/Br/I)	P6	F/Br/I: <1 mg/kg	ND (<1)	47.30	ND (<1)
Beijing ZKGX (Zhongxi)	Combustion-IC (reporting: ND <1 mg/kg for F/Br/I)	P7	F/Br/I: <1 mg/kg	ND (<1)	179.36	ND (<1)
Beijing ZKGX (Zhongxi)	Combustion-IC (reporting: ND <1 mg/kg for F/Br/I)	P8	F/Br/I: <1 mg/kg	ND (<1)	116.94	ND (<1)

Laboratory / Source	Method / Standard	Sample	Reporting limit	F (mg/kg)	Cl (mg/kg)	Br (mg/kg)
Beijing ZKGX (Zhongxi)	Combustion-IC (reporting: ND <1 mg/kg for F/Br/I)	P9	F/Br/I: <1 mg/kg	ND (<1)	62.41	ND (<1)

Section S2. Apparent carbonyl index from ATR-FTIR spectra

To provide a quantitative indicator of oxidation-related functionality, an apparent carbonyl index (CI) was calculated from the ATR-FTIR spectra as CI = A(1714 cm^{-1})/A(1466 cm^{-1}), where absorbance A was computed from transmittance (%T) as A = −log10(T/100). Values were taken at the nearest recorded wavenumbers (1714.5 cm^{-1} and 1465.7 cm^{-1}) for each sample (P1–P9).

Table S4: Apparent carbonyl index derived from ATR-FTIR transmittance spectra (CI = A1714/A1466).

Sample	A1714	A1466	CI (A1714/A1466)
P1	0.0047	0.0537	0.0879
P2	0.0011	0.0604	0.0182
P3	0.0025	0.0696	0.0355
P4	0.0074	0.0728	0.1023
P5	0.0012	0.0443	0.0267
P6	0.0122	0.0697	0.1745
P7	0.0068	0.0979	0.0697
P8	0.0021	0.1070	0.0193
P9	0.0050	0.0793	0.0628

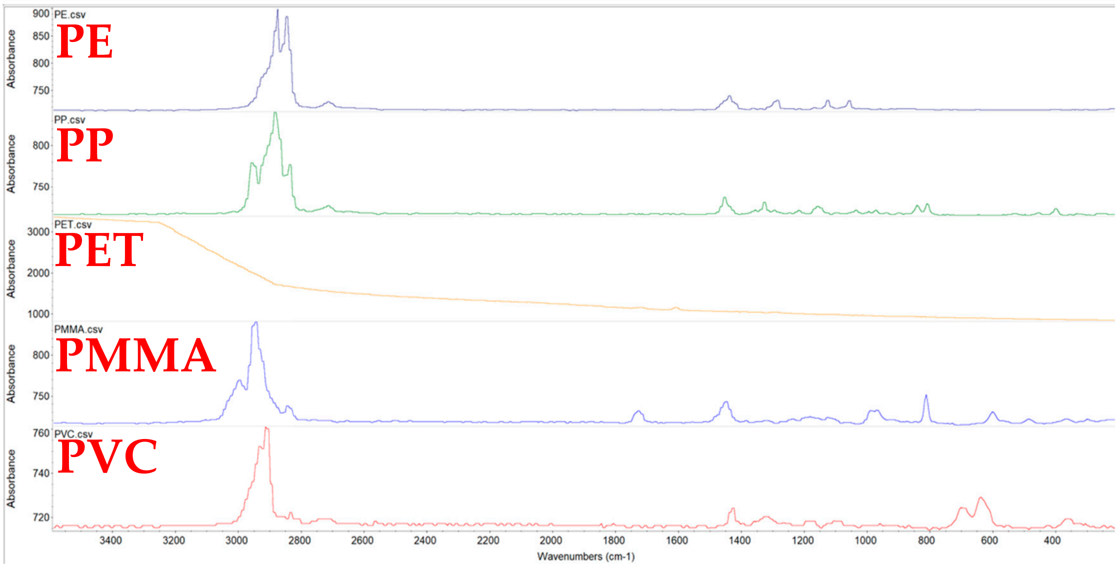

Figure S1: Representative Raman spectra of common thermoplastics: PE, PP, PET, PMMA, and PVC. Each polymer exhibits distinct vibrational band patterns corresponding to its unique chemical structure and functional groups.

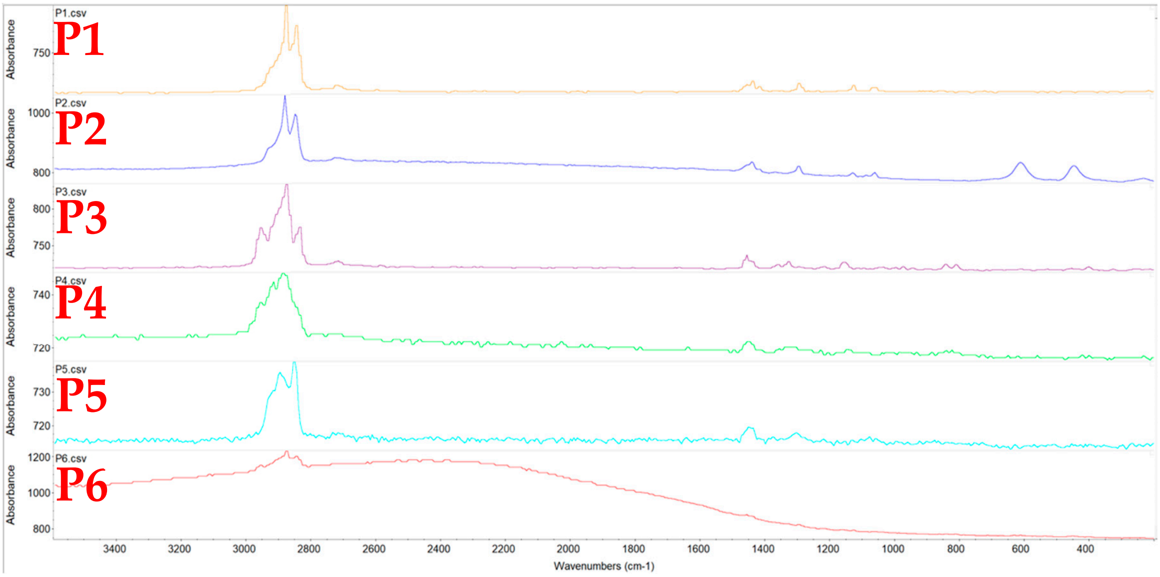

Figure S2: Raman spectra of selected plastic waste samples (P1–P6). P1, P2, and P5 are identified as polyethylene (PE), while P3 corresponds to polypropylene (PP). The P4 spectrum lacks sufficient intensity for identification, likely due to weak Raman scattering. PET and P6 exhibit strong fluorescence interference, which obscures their Raman signals and hinders accurate spectral acquisition. These results illustrate both the utility and limitations of Raman spectroscopy in analyzing certain polymers within complex waste streams.

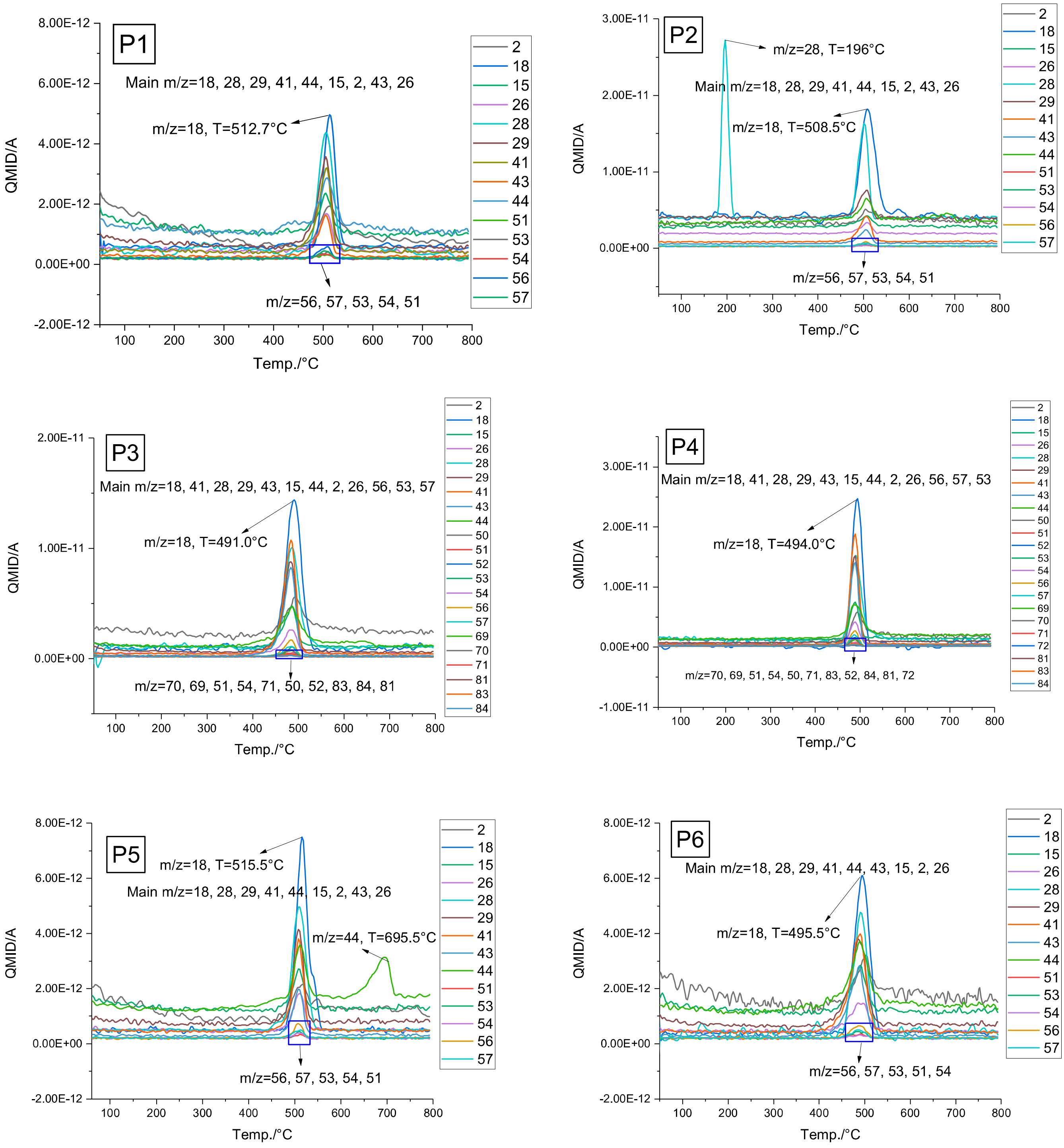

Figure S3: TG–MS profiles of mixed plastic waste samples (P1–P6), showing ion-current signals for selected mass-to-charge (m/z) ratios as a function of temperature.

Table S5: The ion fragments from TG-MS analysis of plastic samples.

m/z	Assigned fragment / species	Interpretation (diagnostic use)
2	H_2	Hydrogen evolution during cracking/dehydrogenation
15	CH_3^+ (fragment)	Hydrocarbon cracking fragments
18	H_2O	Moisture / oxygenated species decomposition
28	C_2H_4	Ethylene—PE-rich cracking signature
29	C_2H_6	Ethane—PE-rich cracking signature
41	$C_3H_5^+$ / C_3H_6 (propylene)	Propylene—PP-rich cracking signature
43	$C_3H_7^+$ / C_3H_8 (propane)	Propane/propyl fragments—PP-rich signature
44	CO_2	Carbonate filler decomposition (e.g., $CaCO_3$) at high temperature

Table S6: The main ion fragments corresponding to the possible compounds.

Fragment(s) observed	Likely source	Notes
m/z 28–29 dominant	PE backbone (β-scission)	Higher relative intensity typically in PE-rich samples
m/z 41–43 dominant	PP backbone cracking	Higher relative intensity typically in PP-rich samples
m/z 44 peak at ~650–700 °C	$CaCO_3$ calcination → CO_2 + CaO	Correlates with elevated ash residue
m/z 18 elevated	Moisture / oxidized polymer segments	May indicate aged/oxidized fractions or absorbed water
Low m/z hydrocarbon suite	Polyolefin thermal cracking	Consistent with expected PE/PP pyrolysis gases

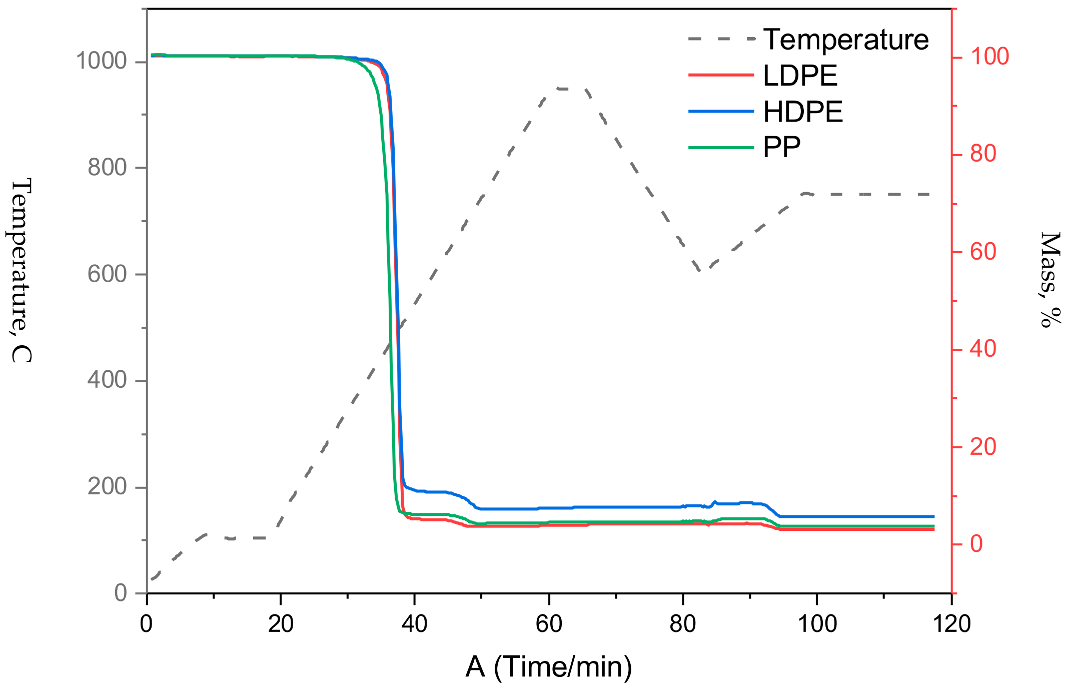

Figure S4: **Thermogravimetric proximate-analysis profiles of samples P7 (LDPE), P8 (HDPE), and P9 (PP).** The **x-axis** shows analysis time during the programmed heating sequence. The **right y-axis** shows the normalized sample mass remaining (**wt%**), while the **left y-axis** shows the furnace temperature (**°C**). The sharp mass-loss region corresponds mainly to volatilization/decomposition, and the final residual plateau is associated with fixed carbon and ash.

References

1. Dahlbo, H., et al., *Recycling potential of post-consumer plastic packaging waste in Finland.* Waste management, 2018. **71**: p. 52-61.
2. Faraca, G. and T. Astrup, *Plastic waste from recycling centres: Characterisation and evaluation of plastic recyclability.* Waste Management, 2019. **95**: p. 388-398.
3. Roosen, M., et al., *Detailed analysis of the composition of selected plastic packaging waste products and its implications for mechanical and thermochemical recycling.* Environmental science & technology, 2020. **54**(20): p. 13282-13293.
4. Eriksen, M.K. and T.F. Astrup, *Characterisation of source-separated, rigid plastic waste and evaluation of recycling initiatives: Effects of product design and source-separation system.* Waste Management, 2019. **87**: p. 161-172.
5. Eriksen, M.K., et al., *Contamination in plastic recycling: Influence of metals on the quality of reprocessed plastic.* Waste management, 2018. **79**: p. 595-606.
6. Hahladakis, J.N., et al., *An overview of chemical additives present in plastics: Migration, release, fate and environmental impact during their use, disposal and recycling.* Journal of hazardous materials, 2018. **344**: p. 179-199.
7. Vijayan, S.K., et al., *Pretreatment of automotive shredder residues, their chemical characterisation, and pyrolysis kinetics.* Sustainability, 2021. **13**(19): p. 10549.

8. Breen, C., et al., *Preparation and characterization of dealuminated metakaolin and its use in the transformation of waste plastics to aromatic hydrocarbons.* Journal of colloid and interface science, 2002. **247**(1): p. 246-250.
9. Dzoh Fonkou, J.P., et al., *Analytical Methods for in-depth assessment of recycled plastics: A review.* Environments, 2025. **12**(5): p. 154.
10. Amin, M.L., et al., *Plastics Recycling: A Comparative Study of Different Analytical Techniques.* Macromolecular Materials and Engineering, 2026. **311**(1): p. e00195.
11. Moreira, L.P., et al., *Raman spectroscopy for identifying postconsumer packaging plastics for recycling.* Journal of the Air & Waste Management Association, 2025. **75**(9): p. 719-734.
12. Akbari Lakeh, M., et al., *Analyzing Sample Size and Cross-Contamination in Plastic Recycling: A Novel Thermal Analysis Method Coupled with Sampling Theory.* ACS Sustainable Chemistry & Engineering, 2025. **13**(19): p. 7179-7188.
13. Sorino, D., et al., *Pyrolysis of mixed contaminated plastic wastes: Assessing the influence of polymers composition, temperature and residence time.* Waste Management, 2025. **201**: p. 114793.
14. Zhou, Z., et al., *Accurate characterization of mix plastic waste using ATR-FTIR spectroscopy and machine learning methods.* PLoS One, 2026. **21**(2): p. e0342178.
